# Development and validation of chest CT-based imaging biomarkers for early stage COVID-19 screening

**DOI:** 10.3389/fpubh.2022.1004117

**Published:** 2022-09-21

**Authors:** Xiao-Ping Liu, Xu Yang, Miao Xiong, Xuanyu Mao, Xiaoqing Jin, Zhiqiang Li, Shuang Zhou, Hang Chang

**Affiliations:** ^1^Department of Pathology, Zhongnan Hospital of Wuhan University, Wuhan, China; ^2^Department of Urology, Zhongnan Hospital of Wuhan University, Wuhan, China; ^3^Key Laboratory of Modern Toxicology of Ministry of Education, School of Public Health, Nanjing Medical University, Nanjing, China; ^4^Department of Radiology, Wuhan Third Hospital, Tongren Hospital of Wuhan University, Wuhan, China; ^5^Department of Emergency, Zhongnan Hospital of Wuhan University, Wuhan, China; ^6^Department of Neurosurgery, Zhongnan Hospital of Wuhan University, Wuhan, China; ^7^Hubei Province Hospital of Traditional Chinese Medicine, Affiliated Hospital of Hubei University of Traditional Chinese Medicine, Hubei Institute of Traditional Chinese Medicine, Wuhan, China

**Keywords:** Coronavirus Disease 2019 (COVID-19), chest CT image, artificial intelligence, imaging biomarker, biomedical imaging application, multicentric retrospective study

## Abstract

Coronavirus Disease 2019 (COVID-19) is currently a global pandemic, and early screening is one of the key factors for COVID-19 control and treatment. Here, we developed and validated chest CT-based imaging biomarkers for COVID-19 patient screening from two independent hospitals with 419 patients. We identified the vasculature-like signals from CT images and found that, compared to healthy and community acquired pneumonia (CAP) patients, COVID-19 patients display a significantly higher abundance of these signals. Furthermore, unsupervised feature learning led to the discovery of clinical-relevant imaging biomarkers from the vasculature-like signals for accurate and sensitive COVID-19 screening that have been double-blindly validated in an independent hospital (sensitivity: 0.941, specificity: 0.920, AUC: 0.971, accuracy 0.931, F1 score: 0.929). Our findings could open a new avenue to assist screening of COVID-19 patients.

## Introduction

Coronavirus Disease 2019 (COVID-19) remains a global pandemic (1, 2). Early detection, early diagnosis, early isolation, and early treatment are essential for the prevention and control of the epidemic. Currently, nucleic acid detection is the most effective tool for COVID-19 diagnosis. However, early COVID-19 detection is still challenging: (1) COVID-19 belongs to a class of highly infectious diseases, with a considerable proportion of patients without obvious clinical symptoms during the onset of disease (2); (2) the critical shortages of resources, including nucleic acid detection kits, also limits the early detection of COVID-19; (3) relatively long time for nucleic acid extraction and detection, non-standard throat swab sampling; (4) relatively high detection cost; (5) false negative rate and limited sensitivity to a certain extent due to relatively low viral load in the early stage of the disease, non-standard throat swab sampling, heterogeneities in types of samples, degradation samples, presence of PCR inhibitors, evolution of the virus, mutations in the viral genome, etc. (3–5); (6) corresponding medical waste (6–8).

Besides the coronavirus etiology, epidemiological contact history, and clinical symptoms, pulmonary imaging, especially chest computed tomography (CT) imaging, plays a unique role for COVID-19 diagnosis (9). For early-stage COVID-19 patients, unifocal ground-glass opacities (GGOs) may present as the main feature, which are most commonly located in the peripheral and inferior lobe. As the disease progresses, these unifocal GGO can develop into multiple GGOs and infiltrate the lungs, while severe consolidation of these lesion may occur in patients with severe disease (10). Lung CT images can be used not only for the diagnosis of COVID-19, but also for assessing the severity of the disease and tracking the lung changes in patients with COVID-19 who have negative nucleic acid tests (11). Several earlier studies showed high sensitivity of CT for the detection of COVID-19, indicating the potential of CT scan in the screening of COVID-19 (4, 12). Fang et al. confirmed in a cohort study of 51 patients with COVID-19 that the detection rate of chest CT for COVID-19 was 98%, while the detection rate of RT-PCR was only 71% (13). At the same time, their study showed that pulmonary vascular prominence as a key feature of COVID-19 can be found in 45–90% of cases. In another cohort study of 1014 patients, Tao et al. (11). compared the detection rate of CT and RT-PCR for COVID-19. In all 1014 patients, RT-PCR and chest CT scans were positive in 59 and 88%, respectively. Among patients with a positive RT-PCR test, chest CT showed a 97% sensitivity for the detection of COVID-19. Among patients with negative RT-PCR results, 75% had positive chest CT results, and 60–93% of cases had positive chest CT results before (or at the same time as) the initial positive RT-PCR result. Before RT-PCR results turned negative, 42% (24/57) of cases showed improvement on follow-up chest CT scans.

However, the CT image characteristics of COVID-19 patients, especially at early stage, are similar to those found in other common pneumonia patients, including those suffering from H7N9 influenza virus pneumonia, mycoplasma pneumonia, chlamydial pneumonia and bacterial pneumonia (14), which requires immediate investigation of potentially underlying characteristics other than the classical ones. Most recently, several interesting studies used artificial intelligence (AI) for the early diagnosis and GGO detection of COVID-19, including PointNet++ (15) and an AI-driven android application (16), where the former can be used for detection and quantifying GGOs in CT scans of COVID-19 patients as well as assessing the severity of the disease, and the latter provided a novel Android application that detected COVID-19 infection from chest CT scans using a highly efficient and accurate deep learning algorithm. Furthermore, neural search architecture network (NASNet)-based algorithm has been demonstrated with great potential in a well-designed computer-aided detection (CAD) system for COVID-19 diagnosis (17). And many other deep learning related systems for COVID-19 detection and diagnosis were summarized in (18). In this study, we developed and validated chest CT-based imaging biomarkers (IBs) for early stage COVID-19 patient (i.e., mild and moderate) screening and differential diagnosis combining Artificial Intelligence (AI) and clinical findings on vascular changes in the lung regions of COVID-19 patients within a system biology approach, which could open a new avenue to assist early stage screening of COVID-19 patients. The major advantages of our imaging biomarkers reside in two folds as follows: (1) they provide robust, accurate and cost-effective COVID-19 screening, which can significantly alleviate the shortage of clinical resources, including both nucleic acid detection kits and experienced radiologists; and (2) they provide a non-invasive diagnostic tool that enables world-wide scalable practical applications. We expect that our imaging biomarkers will be of great significance to reduce the workload of clinicians and to assist in differential diagnosis of COVID-19 from other diseases.

## Materials and methods

### Data collection

The chest CT images in this case-control study were collected from Wuhan Third Hospital (hospital A) and Hubei Provincial Hospital of Traditional Chinese Medicine (hospital B). The inclusion criteria for COVID-19 patients were: (1) patients were diagnosed and confirmed through nucleic acid test from January 2020 to March 2020; (2) patient were with mild or moderate disease status, where the severity was classified according to the Coronavirus Disease 2019 (COVID-19) diagnosis and treatment guideline (trial version 7) (19) issued by the National Health Commission of the People's Republic of China. In addition, both patients with community acquired pneumonia (CAP) and healthy participants (with no obvious abnormalities in chest CT images) were randomly collected from aforementioned two hospitals and used as control groups in training and validation cohorts, independently. The inclusion criteria for control group were: (1) patients who were diagnosed with lung infection on imaging and clinical basis a few months before the onset of the epidemic; (2) patients without severe diseases of respiratory system, cardiovascular or cerebrovascular systems; (3) patients without mental illness or cognitive impairment. This study has been approved by the institutional review board (IRB) of participating hospitals, and been performed according to the required guidelines.

### Imaging protocol for CT chest

Chest CT exams from Hubei Provincial Hospital of Traditional Chinese Medicine were randomly performed with two different scanners: (1) GE Optima 660 CT (GE Healthcare, Milwaukee) and (2) uCT 530 (United imaging, Shanghai), with tube voltage for both scanners at 120 kVp and reconstruction thickness at 0.625 and 1.5 mm, respectively. While, CT exams from Wuhan Third Hospital were performed with GE Discovery CT750 HD (GE Healthcare, Milwaukee) with tube voltage at 120 kVp and reconstruction thickness at 0.625 mm. No intravenous contrast agents were used during scanning in both hospitals.

### Vasculature-like structure enhancement

Blood vessels in lung form tubular structures and the corresponding vasculature-like signal is recognized and enhanced using iterative tangential voting (ITV) (20) within pre-segmented lung regions in 3D, where ITV enforces the continuity and strength of local linear structures and the 3D lung segmentation is achieved *via* level-set method (21). Specifically, each 3D chest CT image is resampled into isotropic image space (voxel size = 1.5 × 1.5 × 1.5 mm) with SimpleITK (version 1.2.4), followed by ITV operating on the isotropic chest CT image gradient information with sigma set to be 0.5 and 1.0 on training and validation cohorts, respectively, to accommodate the technical difference across hospitals.

### Imaging biomarker detection and visualization

We developed an unsupervised feature learning pipeline based on Stacked Predictive Sparse Decomposition (Stacked PSD) (22) for discovery of underlying 3D characteristics from the “vasculature-like signal” space derived by ITV. Given **V=**[**v**_**1**_**, …, v**_**N**_] as a set of 3D “vasculature-like signal” (**N**), the formulation of the imaging biomarker mining pipeline is defined as follows.


$$\begin{array}{l} min_{B,Z,W,G}\;||V-BZ|{|}_{F}^{2}\;+||Z-G\sigma \left(WV\right)|{|}_{F}^{2}\;+\lambda_{1}||Z|{|}_{1}\;\\ \;s.t.\;||b_{i}|{|}_{2\;}^{2}=1,\;\forall i=1,\ldots ,h\; \end{array}$$

where **B=**[**b**_**1**_**, …, b**_**h**_] is a set of imaging biomarkers to be mined (**h**). **Z=**[**z**_**1**_**, …, z**_**N**_] is the sparse biomarker abundance matrix; **W** is the auto-encoder for efficient and effective extraction of sparse biomarker abundance matrix (**Z**) from “vasculature-like signal” (**V**); **G** = *diag*(*g*_1_, .., *g*_*h*_) is a scaling matrix with *diag* being an operator aligning vector, [*g*_1_, .., *g*_*h*_], along the diagonal; **σ**(**·**) is an element-wise sigmoid function; **λ**_1_ is the regularization constant to ensure the sparsity of **Z**, such that only a subset of imaging biomarkers will be utilized during the reconstruction of original “vasculature-like signal.”

The first constraint: ${\left||\text{V}-\text{B}\text{Z}|\right|}_{\text{F}}^{2}$, penalizes the reconstruction error of original “vasculature-like signal” (**V**) with imaging biomarker (**B**) and the corresponding sparse biomarker abundance matrix (**Z**); the second constraint: ${\left||\text{Z}-\text{G}\sigma \left(\text{W}\text{V}\right)|\right|}_{\text{F}}^{2}$, penalizes the approximation error of sparse biomarker abundance matrix (**Z**) with the auto-encoder; the third constraint: ||**Z**||_**1**_, penalizes the sparsity of the biomarker abundance matrix, which helps ensure the utilization/activation of dominant biomarkers during the learning process. The optimization of biomarker pipeline (22) was an iterative process involving ℓ_1_−*minimization* (23) and stochastic gradient descent. Specifically, in this study, we used single network layer with 256 dictionary elements (i.e., patterns) at a fixed patch size of 20 × 20 × 20 voxels and a fixed random sampling rate of 100 3D patches, where the patch size was optimized against reconstruction error and cross-validation performance on training set (Supplementary Figure 15). After training, Stacked PSD reconstructs vasculature-like structures, at given locations, as a combination of pre-trained patterns, with the reconstruction coefficients as the abundance of the corresponding patterns. In training cohort, 8 of 256 patterns were identified with significant correlation with COVID-19 (FDR < 0.05) through cross-validation (training sample rate: 0.8; bootstrap 100 times). The Out of Bag Error (OOB error) was used to measure the prediction error of model on the training set. At last, these 8 significant patterns (i.e., imaging biomarkers) were utilized to build the random forest classification model for COVID-19 screening. A double-blind study was designed and implemented to validate this pre-built model in an independent hospital with three steps: (1) vasculature-like structure enhancement: apply ITV on the isotopically rescaled 3D CT chest scan; (2) imaging biomarker extraction: apply Stacked PSD with pre-identified imaging biomarkers on “vasculature-like signal” space derived from step (1); and (3) double-blind COVID-19 screening: apply the pre-built random forest model on the abundance of pre-identified imaging biomarkers extracted from validation cohort. Visualization of these imaging biomarkers was created in 3D space using ITK-Snap (version 3.8.0), Python (version 3.7.0), Matplotlib (version 3.1.2), Blender (version 2.82) and Three.js (version r115 on GitHub). Snapshots of the three-dimensional visualization were used to generate two-dimensional visualization that overlays with the original CT scans.

### Performance comparison between 3d imaging biomarkers and experienced chest radiologists

We invited two experienced chest radiologists to independently and blindly assess the CT images in our validation cohort, who have 8 and 10 years of clinical imaging diagnosis experience, respectively. And both radiologists have more than 2 months of intense and continuous diagnosis experience of COVID-19 in Wuhan, China. Specifically, de-identified and randomized chest CT images were given to the chest radiologists and their diagnosis were achieved according to their chest CT based clinical practice during COVID-19 diagnosis. Sensitivity and specificity were utilized for performance comparison, with nucleic acid test results as the ground-truth.

### Statistical analysis

The difference in vasculature-like signals and abundance of individual imaging biomarker among different groups were assessed by Mann-Whitney non-parametric test, and association between signatures and COVID-19 were evaluated by logistic regression. The importance of individual imaging biomarker during COVID-19 screening was assessed by random forest package (version 4.6-14) in R (version 3.6.1). Principle component analysis (PCA) and heatmap were performed in R (version 3.6.1) and MATLAB (version 2012b), respectively. The screening performance was characterized with sensitivity, specificity and area under the ROC curve (AUC). Calibration of the screening model was characterized with Hosmer-Lemeshow test in R (version 3.6.1).

## Results

### Study population characteristics

**Figure 1:**
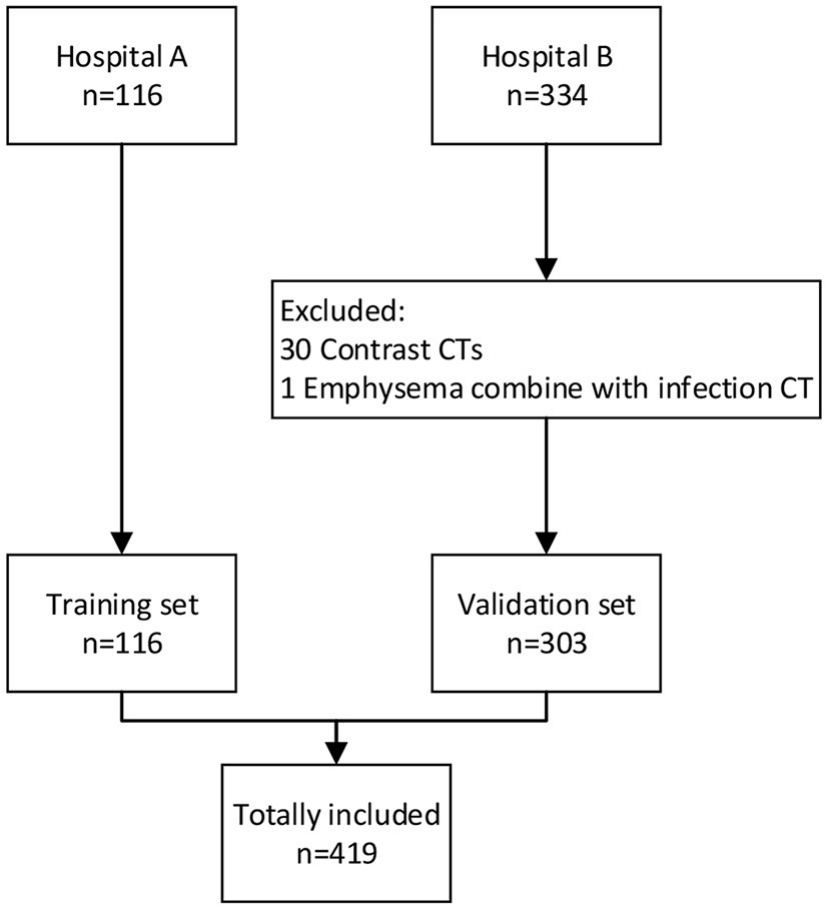
The flowchart for the selection of the participants.

**Figure 2:**
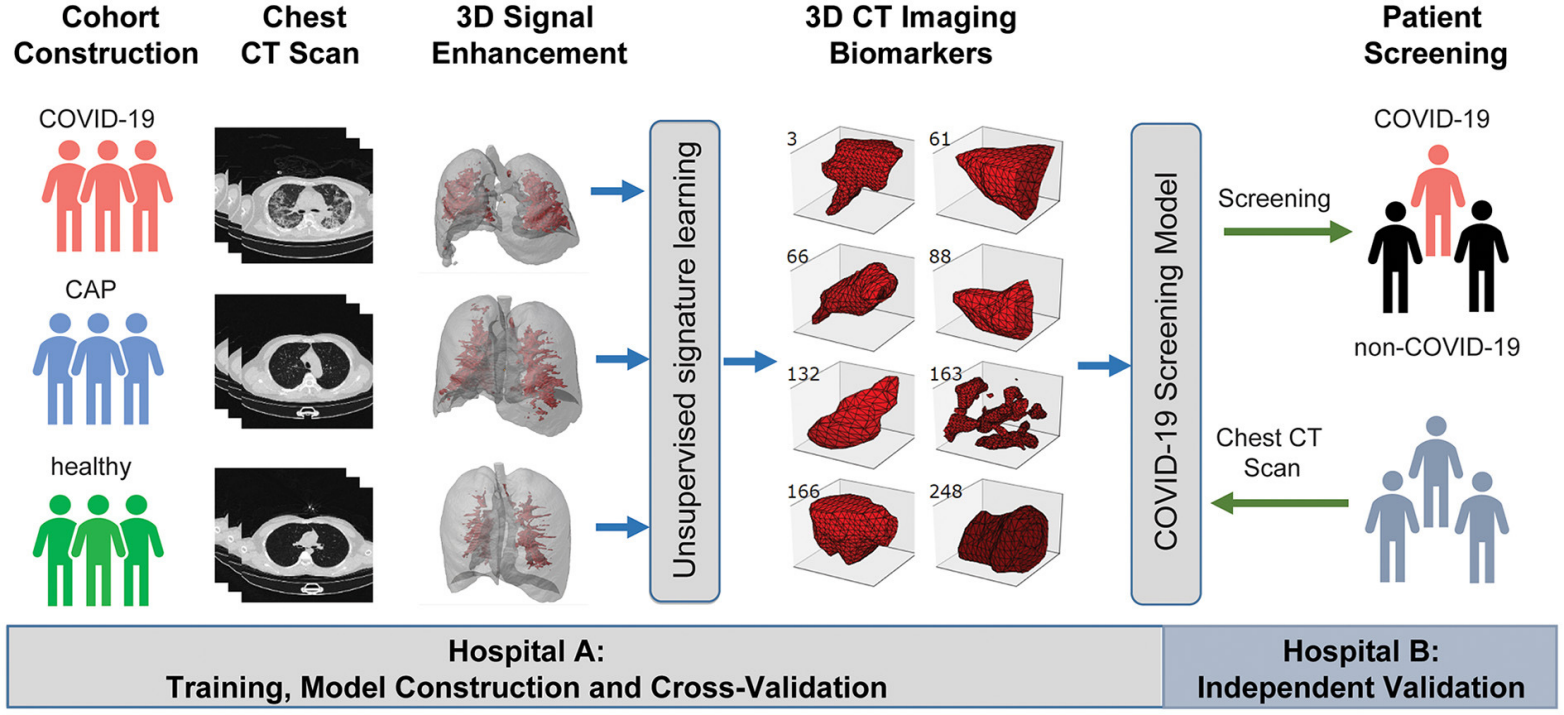
A graphic illustration of the study design. A case-control study was designed to identify chest CT-based imaging biomarkers for COVID-19 patient screening. Biomarker discovery and biomarker-based predictive model construction were conducted using the data from Hospital A (training cohort), which were validated in Hospital B (validation cohort) with the double-blind design.

The flowchart of participant selection in our case-control study was illustrated in Figure 1. The characteristics of cohorts are summarized in Table 1. A total of 419 participants were included in this study. The cohort (*n* = 116) from Hospital A served as training set, the cohort (*n* = 303) from the Hospital B as a double-blind validation set (Figure 2). The median ages of participants in training and validation cohorts were 42 (range: 14–76) and 51 (range: 15–89), respectively. There were 53 (45.7%) females and 63 (54.3%) males in training cohort, and 161 (53.1%) females and 142 (46.9%) makes in validation cohort. Training cohort contained 47 (40.5%) COVID-19 patients, 20 (17.2%) healthy and 49 (42.2%) CAP patients, while validation cohort had 153 (50.5%) COVID-19 patients, 60 (19.8%) healthy, and 90 CAP (29.7%) patients.

**Table 1:** Characteristics of participants included in this study.

**Variables**	**Training**				**Validation**			
	**COVID-19**	**Healthy**	**CAP**	***P*** **-value**	**COVID-19**	**Healthy**	**CAP**	***P*** **-value**
	**(*n* = 47)**	**(*n* = 20)**	**(*n* = 49)**		**(*n* = 153)**	**(*n* = 60)**	**(*n* = 90)**	
**Age** **~Median [Min, Max]**								
	53.0 [31.0, 74.0]	29.0 [14.0, 50.0]	37.0 [16.0, 76.0]	<0.001	64.0 [20.0, 89.0]	41.0 [19.0, 67.0]	38.0 [15.0, 85.0]	<0.001
**Gender**								
Female	24 (51.1%)	7 (35.0%)	22 (44.9%)	0.477	81 (52.9%)	37 (61.7%)	43 (47.8%)	0.247
Male	23 (48.9%)	13 (65.0%)	27 (55.1%)		72 (47.1%)	23 (38.3%)	47 (52.2%)	
**GGO**								
No	6 (12.8%)	20 (100%)	33 (67.3%)	<0.001	12 (7.8%)	60 (100%)	55 (61.1%)	<0.001
Yes	41 (87.2%)	0 (0%)	16 (32.7%)		141 (92.2%)	0 (0%)	35 (38.9%)	
**Consolidation**								
No	43 (91.5%)	20 (100%)	26 (53.1%)	<0.001	123 (80.4%)	60 (100%)	46 (51.1%)	<0.001
Yes	4 (8.5%)	0 (0%)	23 (46.9%)		30 (19.6%)	0 (0%)	44 (48.9%)	

COVID-19, Coronavirus Disease 2019; CAP, community acquired pneumonia; GGO, ground-glass opacities.

### Vasculature-like structure enhancement

**Figure 3:**
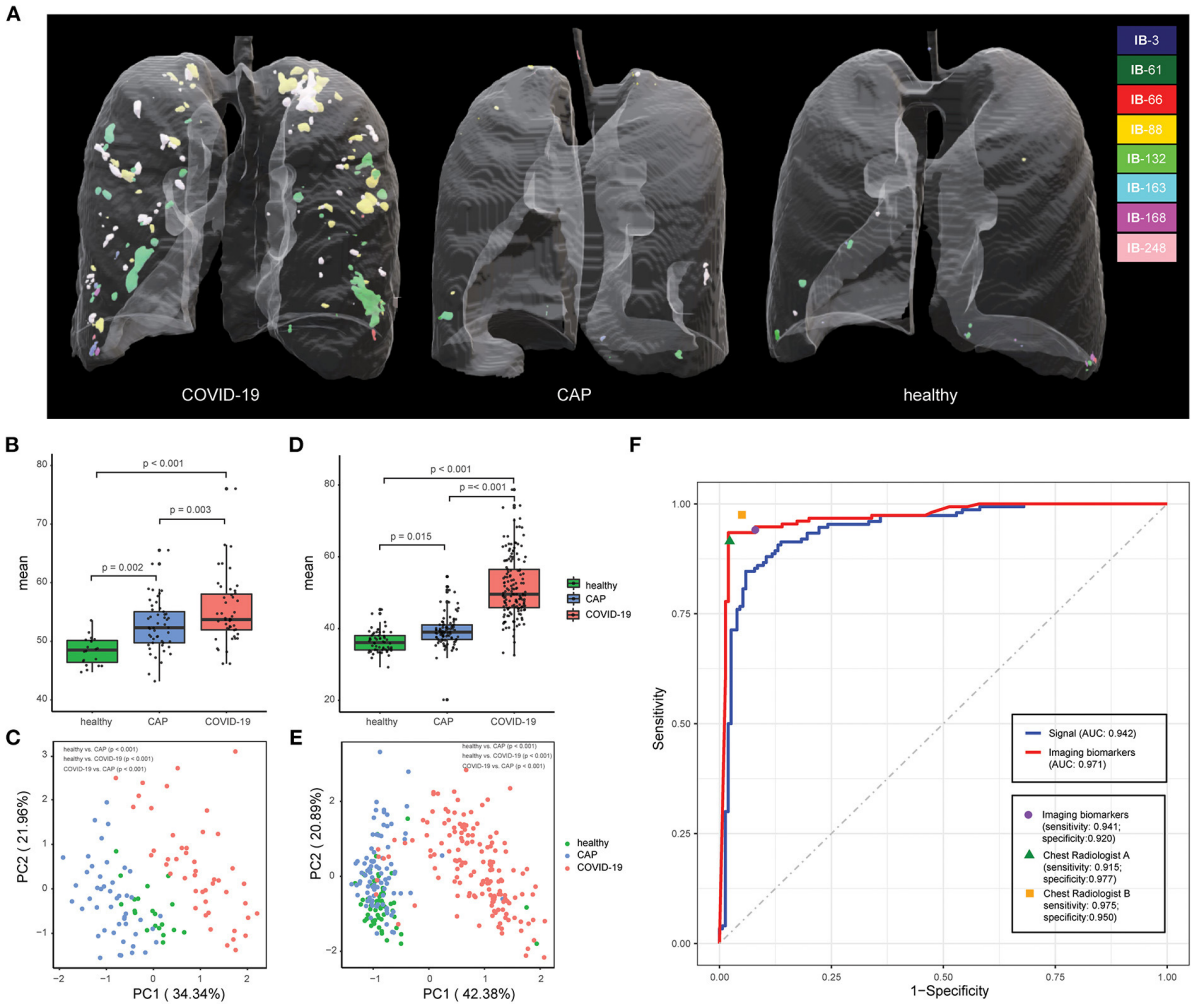
Chest CT-based imaging biomarkers accurately predicts COVID-19. **(A)** Representative examples for 3D multispectral imaging biomarker visualization in COVID-19, CAP and healthy samples. **(B)** The boxplot shows differences in the vasculature-like signals among healthy, community acquired pneumonia (CAP), and COVID-19 patients in the training cohort. The *p*-values were obtained by the non-parametric Mann–Whitney test. **(C)** PCA of 8 imaging biomarkers in the training cohort. Twenty healthy participants (green dots), 49 CAP patients (blue dots), and 47 COVID-19 patients (red dots). The *p*-values were obtained from permutational multivariate analysis of variance (PERMANOVA). **(D)** The boxplot shows differences in the vasculature-like signals among healthy, community acquired pneumonia (CAP), and COVID-19 patients in the validation cohort. The *p*-values were obtained by the non-parametric Mann–Whitney test. **(E)** PCA of 8 imaging biomarkers in the validation cohort. Sixty healthy participants (green dots), 90 CAP patients (blue dots), and 153 COVID-19 patients (red dots). The *p*-values were obtained from permutational multivariate analysis of variance (PERMANOVA). **(F)** Screening performance of signal-based model, imaging biomarker-based model, and two COVID-19 experienced radiologist on validation cohort.

**Figure 4:**
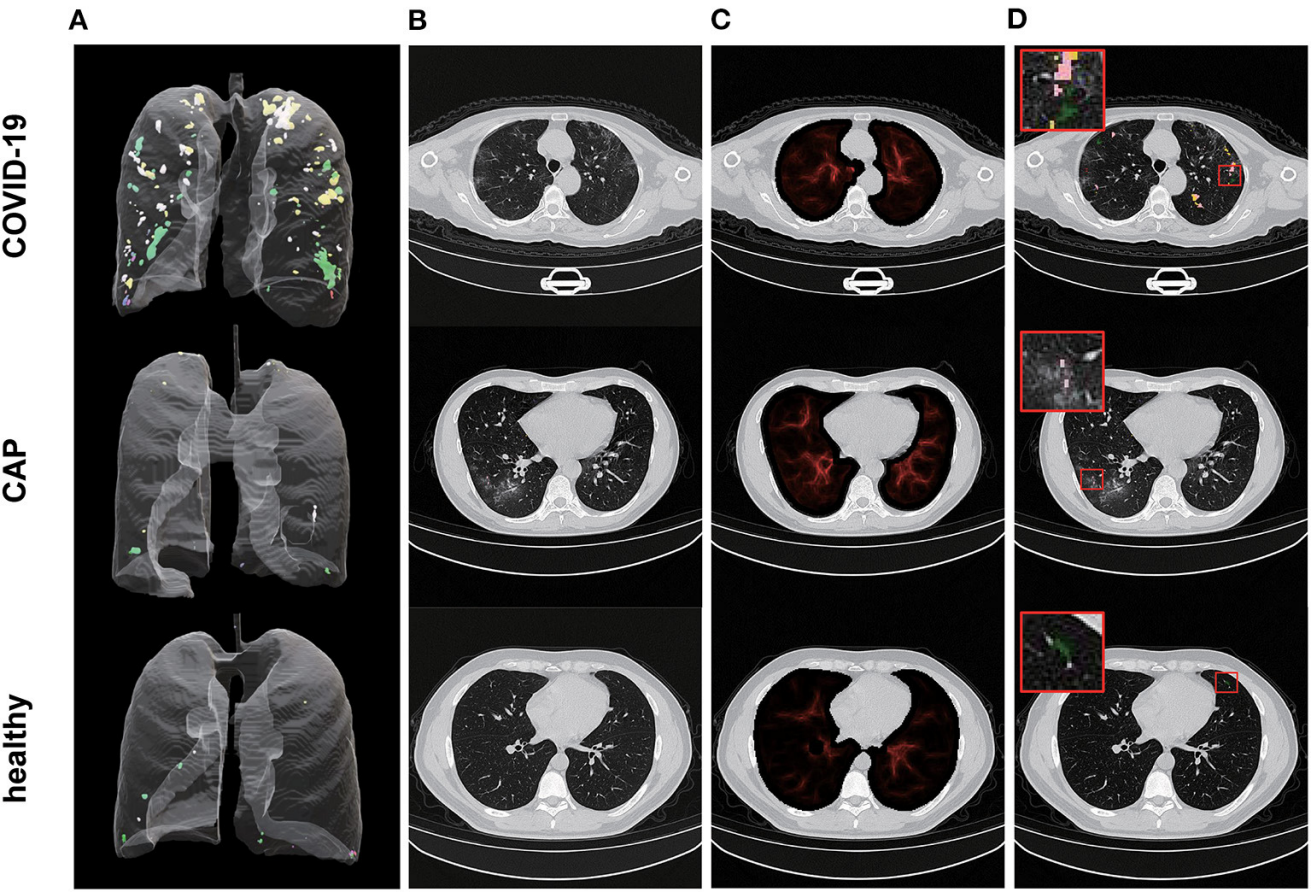
Illustration of representative CT image and the corresponding vasculature-structure enhancement and multi-spectral staining in COVID-19, CAP and healthy samples. **(A)** Representative examples for 3D multispectral imaging biomarker visualization (3D animations are provided by Supplementary Videos 4–6); **(B)** Representative 2D CT images; **(C)** Corresponding 2D vasculature-structure enhancement (enhancement for entire chest CTs are provided by Supplementary Videos 1–3); **(D)** Corresponding 2D multi-spectral staining (2D multi-spectral staining for entire chest CTs are provided by Supplementary Videos 7–9).

Inspired by recent findings on vascular changes in lung tissue of COVID-19 patients, including vascular congestion/enlargement, small vessels hyperplasia and vessel wall thickening (24–26), we hypothesize that, compared with healthy and CAP patients, COVID-19 patients have significantly more vascular changes in the lung. Therefore, we built a machine learning pipeline on enhanced vasculature-like structures formed by blood vessels to discover underlying characteristics from chest CT of early stage COVID-19 patients. Specifically, the vasculature-like structure was recognized and enhanced with ITV (20) in both training and validation cohorts as a pre-processing step. Interestingly in training cohort, the mean vasculature-like signal (i.e., the average intensity of vasculature-like structures recognized and enhanced by ITV in lung region) reveals significant differences (*p* < 0.05) between healthy, CAP and COVID-19 patients (Figure 3B). Examples of vasculature-like structure enhancement are illustrated in Figures 4A–D and Supplementary Videos 1–3 for COVID-19, CAP, and healthy cases, respectively. These findings are not only consistent with the clinical observations (24–26), but also leads to remarkable differentiation between COVID-19 and non-COVID-19 groups in training cohort [AUC = 0.721 (95% CI (0.536, 0.861)), Supplementary Figure 1, blue curve] with logistic regression. Altogether, it encourages us to identify imaging biomarkers from the “vasculature-like signal” space to assist accurate early stage COVID-19 screening.

### Imaging biomarker detection and COVID-19 screening

**Figure 5:**
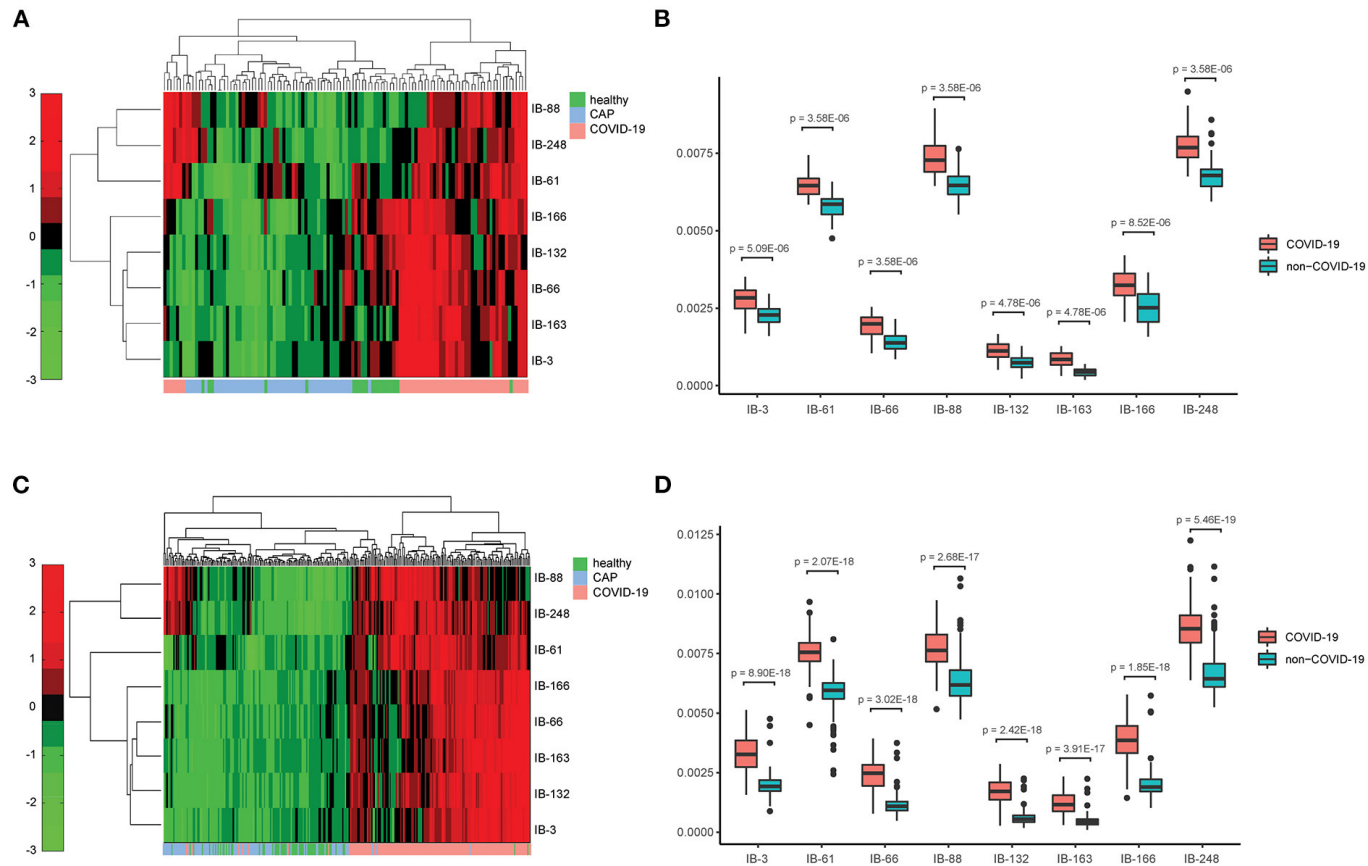
Chest CT-based imaging biomarkers provide consistent and significant distinction between COVID-19 patients and others across hospitals. **(A)** Heatmap of the relative abundance of imaging biomarkers shows distinct clusters with respect to COVID-19 and non-COVID-19 groups in training cohort; **(B)** Individual imaging biomarker shows significantly higher relative abundance in COVID-19 patients in training cohort; **(C)**. Heatmap of the relative abundance of imaging biomarkers shows distinct clusters with respect to COVID-19 and non-COVID-19 groups in validation cohort; **(D)** Individual imaging biomarker shows significantly higher relative abundance in COVID-19 patients in validation cohort.

Next, we applied Stacked PSD (22) on the “vasculature-like signal” space from training cohort. Two hundred fifty-six dictionary elements were learned and optimized, where 8 of them have significant positive correlations with COVID-19 (FDR < 0.05, Supplementary Tables 1, 2, Supplementary Figures 2, 3). These eight COVID-19-relevant signatures (i.e., imaging biomarkers, Figure 2 3D CT Imaging Biomarkers panel, and Supplementary Figures 4, 5) allow the construction of multispectral staining in the entire lung region (Figure 3A), which is further demonstrated in 3D (Supplementary Videos 4–6) and 2D (Supplementary Videos 7–9) animations. The 8 imaging biomarkers clearly separate COVID-19 patients from others in training cohort by PCA (Figure 3C) and clustering (Figure 5A) analysis, where each individual biomarker has significantly different abundance between COVID-19 patients and others (Figure 5B). Finally, we built a random forest classification model for COVID-19 screening based on these imaging biomarkers within training cohort [the OOB error = 3.26%, 95% CI (1.09–6.52%); AUC = 1.000, 95% CI (0.982, 1.000); Sensitivity = 1.000, 95% CI (0.800, 1.000); Specificity = 1.000, 95% CI (0.930, 1.000); F1 score = 0.966, 95% CI (0.923, 1.000); accuracy = 0.964, 95% CI (0.900, 1.000); precision = 1.000, 95% CI (0.875, 1.000); Supplementary Figure 1, red curve]. Additionally, we show that each individual imaging biomarker contribute differently during screening, where IB-163 played the most important role (Supplementary Figure 1b), with the best single biomarker performance [AUC = 0.893, 95% CI (0.842, 0.953), Supplementary Figures 1c, 6, Supplementary Table 3].

### Double-blind test of imaging biomarkers in validation cohort

The vasculature-like structure enhancement process was applied onto validation cohort, followed by biomarker extraction. As seen in training cohort, we observed the distinction of mean vasculature-like signal between different groups (Figure 3D). The logistic regression model pre-built on training cohort with mean vasculature-like signal led to accurate prediction in validation cohort (AUC = 0.942, Figure 3F, blue curve). The combination of 8 pre-identified imaging biomarkers also clearly separates the COVID-19 patients from others in validation cohort (Figures 3E, 5C), where each individual biomarker consistently revealed significantly different abundance (Figure 5D). Excitingly, we found the pre-built random forest model based on pre-obtained imaging biomarkers predict COVID-19 with excellent sensitivity (0.941), specificity (0.920), accuracy (0.931), precision (0.939), F1 score (0.929), and AUC (0.971), which is competitive with two COVID-19 experienced chest radiologists (Figure 3F): radiologist A (sensitivity = 0.915; specificity = 0.977, accuracy = 0.944, precision = 0.898, F1 score = 0.946, radiologist B (sensitivity = 0.975; specificity = 0.950, accuracy = 0.974, precision = 0.973, F1 score = 0.973). In addition, the competitiveness is further demonstrated using bootstrapping strategy (100 iterations, 80% sampling rate) on various performance metrics between imaging biomarkers and two radiologists (Supplementary Figure 7). Furthermore, the Hosmer-Lemeshow test suggested no departures from perfect fit on both training (*p* = 0.867) and validation (*p* = 1.000) cohorts (Supplementary Figure 8).

### Case study

**Figure 6:**
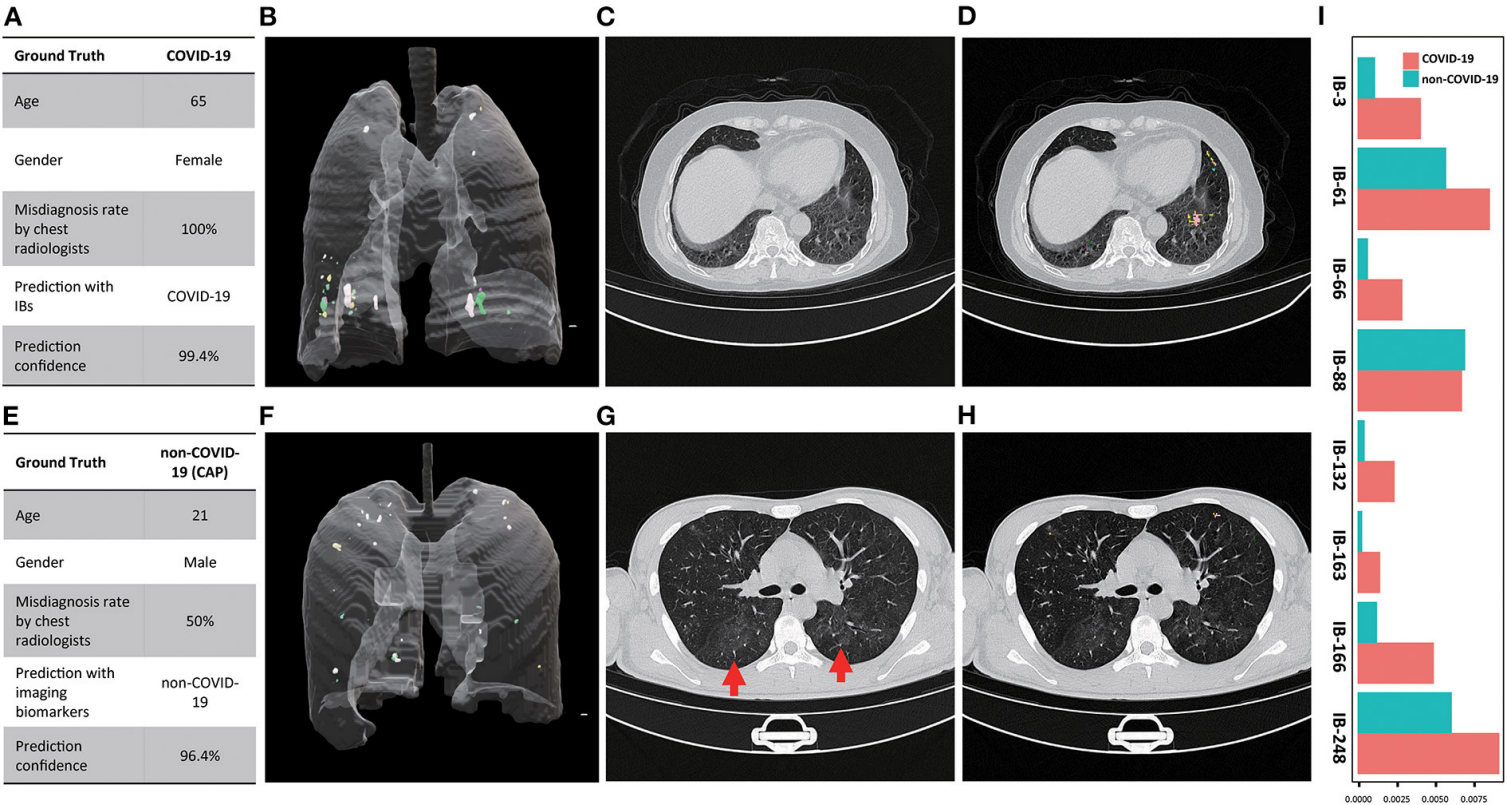
Examples of misdiagnosed cases by participating chest radiologist(s). **(A)** Characteristics of the COVID-19 patient and the corresponding diagnosis (chest radiologists)and screening (imaging biomarkers) results; **(B)** 3D multi-spectral staining of the COVID-19 patient (3D animation can be found in Supplementary Video 10); **(C)** Representative CT image slice of the COVID-19 shows no typical abnormity related to COVID-19, which led to the false negative decision of both chest radiologist; **(D)** the corresponding 2D multi-spectral staining of the selected CT image slice (2D animation of the entire CT scan can be found in Supplementary Video 12); **(E)** Characteristics of the CAP patient and the corresponding diagnosis and screening results; **(F)** 3D multi-spectral staining of the CAP patient (3D animation can be found in Supplementary Video 11); **(G)** Representative CT image slice of the CAP patient shows the typical while subtle image characteristics (GGO, marked by red arrows) of the COVID-19 in the upper lobe of both lungs, which led to the false positive decision by one of the chest radiologists; **(H)** The corresponding 2D multi-spectral staining of the selected CT image slice (2D animation of the entire CT scan can be found in Supplementary Video 13); **(I)** Relative abundance of imaging biomarkers differentiate the COVID-19 from CAP patient.

We further examined the capability of our imaging biomarkers with misdiagnosed cases by our participating radiologists, where a COVID-19 patient (female, 65 years old, Figure 6A), and a CAP patient (male, 21 years old, Figure 6E) were included. Due to the lack of typical abnormality (Figure 6C, both experts misdiagnosed the COVID-19 patient. Meanwhile, the CAP patient showed subtle misleading characteristics (i.e., GGO) in the upper lobe of both lungs (Figure 6G, red arrows), and led to false positive decision by one of the experts. Obviously, in real-world clinical practice, chest CT based early screening of COVID-19 can be challenging for both clinical experts, and typical-abnormality-driven end-to-end AI systems, due to either lack of typical abnormality in COVID-19 cases or presence of misleading characteristics in non-COVID-19 cases. In contrast, our imaging biomarkers provided both perceptual (Figure 6B vs. Figure 6F, Supplementary Video 10 vs. Supplementary Video 11; Figure 6D vs. Figure 6H, Supplementary Video 12 vs. Supplementary Video 13) and quantitative (Figure 6I) distinctions (except for IB-88) for these ambiguous cases, and therefore enables accurate screening with high confidence (Figures 6A,E; over 96% confidence for both cases).

### Further comprehensive justification of the robustness of imaging biomarkers

We (1) switched the role of two hospitals with Hospital B as training cohort and A as validation cohort [sensitivity: 0.957, specificity: 0.841, accuracy: 0.888, precision: 0.951, F1 score: 0.892 and AUC: 0.961 (95% CI (0.932, 0.994))]; and (2) combined two cohorts for cross-validation with random training sample rate at 80% and 100 bootstrap iterations [Supplementary Table 4, Supplementary Figure 9; sensitivity: 0.950 (95% CI (0.875, 1.000)), specificity: 0.977 (95% CI (0.909, 1.000)), accuracy: 0.953 (95% CI (0.909, 0.995)), precision: 0.973 (95% CI (0.902, 1.000)), F1 score: 0.951 (95% CI (0.903, 0.994)) and AUC: 0.980 (95% CI (0.937, 0.999))], which further demonstrated the robustness of our imaging biomarkers. Also, we performed age-group-wised (<60 and ≥60 years old) study (27) on combined cohorts to evaluate the age impact on our imaging biomarkers. As shown in Supplementary Table 5, age was comparable between the two groups both in training and validation set in ≥60 years old groups. It is clear that (Supplementary Figure 10), for all signatures (except IB-88), (1) within all age groups, the imaging biomarker has significantly higher abundance in COVID-19 patients; (2) across age groups, the imaging biomarker has significant higher abundance in category (COVID-19, <60 years old) than in category (non-COVID-19, ≥60 years old). Additionally, correlation analysis (Supplementary Table 6, Supplementary Figure 11) revealed (1) statistically non-significant (FDR > 0.05) “poor correlation” (28) between age and single/imaging biomarkers within COVID-19 group; and (2) three statistically significant (FDR < 0.05) “poor/fair correlation” (28) between age and (IB-3, IB-61, and IB-166) within Non-COVID-19 group. Also, we investigated the abundance of imaging biomarkers between age groups on both training and validation sets (Supplementary Figure 12), and confirmed that most biomarkers were significantly different between COVID-19 and non-COVID-19 age groups on both training and validation sets, except for IB-61, IB-88 and IB-248, potentially due to the limited sample numbers in each age group. In addition, we showed that the prediction model built upon our 8 biomarkers and patient age yielded statistically identical performance compared to the original prediction model with our 8 biomarkers only on training cohort (Supplementary Table 7, Supplementary Figure 13; *p* > 0.05; 100 bootstrap iterations with random training sample rate at 80%), which was further confirmed by the quantitative evaluation of these two pre-built models on validation cohort (Supplementary Table 8, Supplementary Figure 14). These evidences indicate that age does not impact our imaging biomarker nor the corresponding screening model.

### Potential underlying molecular and biological mechanisms

Severe acute respiratory syndrome coronavirus 2 (SARS-CoV-2) infection triggers a reverse host immunity response, followed by propagation of the virus especially to the ACE2 rich organs, among which lungs remain to be the mostly affected organ resulting in severe respiratory disease in many individuals. Also, the unrestrained immune response triggers lung inflammation with unfavorable outcomes, where reactive oxygen species (ROS) are key signaling molecules with an important role in the progression of inflammatory disorders (29). Recent studies on SARS-CoV-2 revealed the potential molecular and biological mechanisms strikingly similar to what have been seen in pulmonary vascular disease development, including inflammation, hypoxia, oxidative stress, and DNA damage, that contribute to the promotion of endothelia dysfunction, vascular leak, and pulmonary microthrombi (30–36). Furthermore, SARS-CoV-2 leads to cytokine outburst, including IL-6, IL-1b, IL-2, IL-10, and monocyte chemoattractant protein-1 (MCP-1), which are also associated with vascular dysfunction and vascular disease such as atherosclerosis, abdominal aortic aneurysm, varicose veins and hypertension (37). Consequently, the SARS-CoV-2-related disease (COVID-19) revealed significant effects on the lungs and the pulmonary vasculature. In addition to parenchymal abnormalities, pulmonary microthrombi, ventilation-perfusion mismatch, and hypoxemia are also observed which are due to disseminated intravascular coagulation, endothelial dysfunction, and impaired hypoxic pulmonary vasoconstriction. Importantly, our findings are consistent with these molecular- and biological-driven effects on pulmonary vasculature, which provides the underlying molecular and biological mechanism for our imaging biomarkers. Furthermore, our study indicates that these molecular and biological effects on pulmonary vasculature exist and can be quantitative captured even at the early stage of COVID-19. With above molecular and biological potentials, we believe our imaging biomarkers could help assess the severity as well as the treatment outcome of COIVD-19 patients.

## Discussion

In this study, we developed and validated chest CT based 3D imaging biomarkers for early stage COVID-19 screening. We suggest, compared to healthy and CAP patients, COVID-19 patients may have significantly more vascular changes in lung tissue (24–26), which leads to the discovery of robust imaging biomarkers for early stage COVID-19 screening. Our double-blind validation across hospitals and CT scanners confirms (1) the hypothesis on the quantitative difference of vascular changes among COVID-19 and non-COVID-19 groups; (2) the robustness and effectiveness of our imaging biomarkers in real-world clinical settings with considerable technical variations; and (3) the competitiveness with COVID-19 experienced chest radiologists. Detailed case study further demonstrates the capability of our imaging biomarkers especially for ambiguous cases, which is common during early-stage COVID-19 screening. Further comprehensive evaluation suggests our imaging biomarkers are independent from hospital (batch effect free) and age (independent value). In addition, the robustness and effectiveness of our vasculature-related imaging biomarkers attribute to the effects of COVID-19 on the lungs and the pulmonary vasculature, including pulmonary microthrombi, ventilation-perfusion mismatch and hypoxemia, which are resulted from the potential mechanisms of SARS-CoV-2, including inflammation, hypoxia, oxidative stress, and DNA damage, that contribute to the promotion of endothelia dysfunction, vascular leak, and pulmonary microthrombi. For example, the structure of our best performing single imaging biomarker: IB-163 (Figure 2), potentially resembles the phenomenon related to vascular leak.

Specifically, our demonstrated screening capability was built upon biomedical evidence, robustness, interpretability, scalability, and accuracy to maximize its clinical impact. Different from many existing end-to-end solutions (38), our work was realized by seamless integration of the blood-vessel-related clinical insights within an highly compact and scalable unsupervised learning framework with feed-forward biomarker extraction strategy involving only element-wise non-linearity and matrix multiplication (22), which helped alleviating challenges due to the (1) absence or subtle typical abnormal characteristics in chest CT especially for early stage COVID-19 patients; (2) presence of misleading characteristics in chest CT from non-COVID-19 cases; and (3) requirement of large training cohort and excessive computational resources by many end-to-end AI models. Subsequently, it enables the discovery of robust biomedical-relevant imaging biomarkers effectively from a small training cohort (*n* = 116), and thereafter scalable [~50 s *via* Matlab with Intel(R) Xeon(R) CPU E5-2630 v3], superior and stable screening performance.

The major limitation of our study is the exclusion of non-image information, including clinical symptoms and laboratory findings, which are valuable for COVID-19 diagnosis (39, 40). However, given (1) our current focus on imaging biomarker development and validation, and (2) the nature of biomarker detection and utilization (different from end-to-end AI systems), it is straightforward to combine non-image information with our imaging biomarkers to realized multi-modality screening capability *via* scalable techniques (e.g., random forest). Additionally, the CAP patients included in this study were from patients with pneumonia before the outbreak, which were clinically diagnosed (based on imaging findings) and treated with empirical drugs. Therefore, like many retrospective studies (38, 39, 41), the CAP patients cannot be classified according to specific pathogens, which requires a future prospective study. Chest CT scan also has certain shortcomings: first, similar to RT-PCR, chest CT scan also has certain false negative rates when the viral load is relatively low. Second, lung CT imaging is relatively expensive compared to RT-PCR testing, which may limit its use in less developed areas. Third, if the lung CT scan environment is not sufficiently disinfected, it may cause cross-infection among the tested persons. In the early stage of this epidemic, due to the high false negative rate of RT-PCR and the long return time of the test results, the chest CT scan has made up for the shortcomings of RT-PCR, and a large number of patients have been timely diagnosed, isolated and treated (42, 43). Even with the improvement of RT-PCR detection technology, chest CT still remains useful for auxiliary diagnosis and assessment of disease severity and prognosis (44–47), as well as for its potential screening capability in consideration of the possible variation of the virus during RT-PCR test. We also realized that the accessibility of CT scanner may potentially impact the utilization of our findings. However, given the (1) the demonstrated clinical implications; and (2) the prognostic potential of our imaging biomarkers combining with clinical information, we strongly believe the potential of our study in providing a valuable alternative besides nucleic acid toolkit for early-stage COVID-19 screening with world-wide impact.

To summarize, COVID-19 epidemic is a world-wide threat (48), consuming the medical resources in some countries (49). Facing the short supply of nucleic acid detection kits in many countries, most chest CT based computational studies were built upon typical abnormity in an end-to-end fashion, which can suffer due to the lack/subtle amount of such typical characteristics in early stage COVID-19 patients, or even misleading characteristics in others. To overcome these challenges, we identified robust imaging biomarkers from vasculature-like signal in chest CT scans for accurate early stage COVID-19 screening with major advantages as follows: (1) they provide robust, accurate and cost-effective COVID-19 screening, which can significantly alleviate the shortage of clinical resources, including both nucleic acid detection kits and experienced chest radiologists; and (2) they provide a non-invasive diagnostic tool that enables world-wide scalable practical applications. Our merits originate from the system biology approach, and thus provide important clinical insights/knowledge that is beyond existing clinical practice as well as the capability/scope of many existing end-to-end AI systems. As future work, our imaging biomarkers may (1) be combined with non-image information to improve screening performance; and (2) facilitate the prediction of COVID-19 patients' prognosis and clinical outcome at early stage.

## Data availability statement

The original contributions presented in the study are included in the article/Supplementary material, further inquiries can be directed to the corresponding author.

## Ethics statement

The studies involving human participants were reviewed and approved by Wuhan Third Hospital; and Hubei Provincial Hospital of Traditional Chinese Medicine. Written informed consent for participation was not required for this study in accordance with the national legislation and the institutional requirements.

## Author contributions

HC, ZL, MX, and SZ designed the study. X-PL, XM, XY, XJ, and HC performed the analysis. HC, ZL, and X-PL wrote the manuscripts. All authors revised the manuscript. All authors contributed to the article and approved the submitted version.

## Conflict of interest

The authors declare that the research was conducted in the absence of any commercial or financial relationships that could be construed as a potential conflict of interest.

## Publisher's note

All claims expressed in this article are solely those of the authors and do not necessarily represent those of their affiliated organizations, or those of the publisher, the editors and the reviewers. Any product that may be evaluated in this article, or claim that may be made by its manufacturer, is not guaranteed or endorsed by the publisher.
