# Supplementary material for: Development and validation of chest CT-based imaging biomarkers for early stage COVID-19 screening
Source: Front Public Health. 2022 Sep 21;10:1004117. doi: 10.3389/fpubh.2022.1004117 (PMC9533142; doi:10.3389/fpubh.2022.1004117)
Supplement: Supplementary file 1 [file Table_1.docx]

**Supplementary Table 1** Significant dictionary elements (imaging biomarkers) positively correlated with COVID-19.

| Dictionary element | OR | 95% CI | | P value | FDR |
| --- | --- | --- | --- | --- | --- |
|  |  | Lower | Upper |  |  |
| IB-3 | 7.71E+36 | 7.7352E+18 | 2.26E+57 | 0.00015289 | 0.000430108 |
| IB-61 | 3.25153E+18 | 359.9820936 | 2.60E+35 | 0.026274294 | 0.0431216 |
| IB-66 | 9.30E+46 | 3.97E+27 | 2.08E+69 | 8.61E-06 | 4.24E-05 |
| IB-88 | 1.37E+21 | 13586381.21 | 3.57E+36 | 0.004540289 | 0.008673985 |
| IB-132 | 4.12E+73 | 8.99E+44 | 1.32E+107 | 2.94E-06 | 1.79E-05 |
| IB-163 | 4.27E+151 | 6.10E+101 | 3.47E+214 | 1.08E-07 | 2.55E-06 |
| IB-166 | 3.09E+28 | 1.71393E+15 | 3.88E+43 | 7.19E-05 | 0.000224565 |
| IB-248 | 3.55897E+18 | 5413396.828 | 5.74E+31 | 0.003411235 | 0.006769584 |

Abbreviation: IB, Imaging biomarker; OR, Odds ratio; FDR, False discovery rate; CI, Confidence interval.
