# Supplementary material for: Development and validation of chest CT-based imaging biomarkers for early stage COVID-19 screening
Source: Front Public Health. 2022 Sep 21;10:1004117. doi: 10.3389/fpubh.2022.1004117 (PMC9533142; doi:10.3389/fpubh.2022.1004117)
Supplement: Supplementary file 2 [file Table_2.docx]

| **Supplementary Table 2** Associations between dictionary elements and COVID-19 | | | | | |
| --- | --- | --- | --- | --- | --- |
| Dictionary element | OR | 95%CI | | P value | FDR |
|  |  | Lower | Upper |  |  |
| IB-1 | 0.02782051 | 7.74E-13 | 733398190.2 | 0.769389069 | 0.820681674 |
| IB-2 | 1.20E-19 | 6.76E-33 | 1.63E-07 | 0.003254665 | 0.006560584 |
| IB-3 | 7.71E+36 | 7.7352E+18 | 2.26E+57 | 0.00015289 | 0.000430108 |
| IB-4 | 1.38E-16 | 9.47E-28 | 9.60E-07 | 0.002796479 | 0.005681735 |
| IB-5 | 7.83E-35 | 2.29E-51 | 1.64E-20 | 1.28E-05 | 5.96E-05 |
| IB-6 | 1.17E-19 | 3.61E-30 | 2.76E-10 | 0.000172671 | 0.00047531 |
| IB-7 | 8.48E-72 | 7.89E-101 | 9.84E-49 | 6.77E-08 | 2.39E-06 |
| IB-8 | 2.97E-16 | 1.31E-30 | 0.008347471 | 0.027487444 | 0.044820292 |
| IB-9 | 8.23E-12 | 7.27E-35 | 2.17091E+11 | 0.336070217 | 0.409685597 |
| IB-10 | 7.68E-54 | 7.98E-78 | 5.27E-34 | 1.76E-06 | 1.22E-05 |
| IB-11 | 7.25E-15 | 6.07E-26 | 9.05E-05 | 0.008344661 | 0.014938693 |
| IB-12 | 1.51E-33 | 1.51E-50 | 3.03E-19 | 3.60E-05 | 0.000131746 |
| IB-13 | 8.01E-29 | 6.93E-43 | 1.69E-16 | 2.66E-05 | 0.000103044 |
| IB-14 | 4.45E-31 | 1.99E-47 | 1.02E-16 | 9.52E-05 | 0.000280126 |
| IB-15 | 2.44E-25 | 5.83E-40 | 2.70E-12 | 0.000448447 | 0.001074152 |
| IB-16 | 8.622085186 | 8.21E-15 | 1.36754E+16 | 0.902648238 | 0.928023891 |
| IB-17 | 0.263842859 | 9.28E-17 | 4.29697E+14 | 0.940390397 | 0.951541271 |
| IB-18 | 1.50E-25 | 8.70E-42 | 3.43E-11 | 0.00134661 | 0.002825674 |
| IB-19 | 6.05E-09 | 9.95E-20 | 104.6753722 | 0.122792268 | 0.166321802 |
| IB-20 | 3.65E-26 | 1.40E-39 | 1.54E-14 | 6.11E-05 | 0.000193301 |
| IB-21 | 6.29E-35 | 9.39E-52 | 1.14E-20 | 1.39E-05 | 6.24E-05 |
| IB-22 | 2.84E-27 | 4.02E-47 | 2.88E-09 | 0.005575256 | 0.010417997 |
| IB-23 | 2.32E-16 | 2.21E-32 | 0.243957544 | 0.046825681 | 0.072650754 |
| IB-24 | 1.04E-56 | 3.61E-81 | 2.99E-36 | 8.30E-07 | 6.86E-06 |
| IB-25 | 3.12E-17 | 2.13E-32 | 0.003178695 | 0.025359458 | 0.042431511 |
| IB-26 | 8.30E-55 | 9.92E-79 | 2.74E-35 | 8.93E-07 | 7.14E-06 |
| IB-27 | 0.012930157 | 2.52E-21 | 6.88829E+16 | 0.842317822 | 0.887380093 |
| IB-28 | 3.25E-14 | 2.55E-30 | 94.0240192 | 0.091856816 | 0.129205192 |
| IB-29 | 5.01E-24 | 1.23E-38 | 2.49E-11 | 0.000741299 | 0.001664671 |
| IB-30 | 28170.24127 | 2.68E-09 | 5.94856E+17 | 0.504736104 | 0.574277523 |
| IB-31 | 1.55E-21 | 4.94E-35 | 4.73E-09 | 0.001585039 | 0.00327234 |
| IB-32 | 8.20E-21 | 1.47E-35 | 4.03E-07 | 0.005328245 | 0.010029638 |
| IB-33 | 1.12E-29 | 1.02E-44 | 1.54E-16 | 4.96E-05 | 0.000168068 |
| IB-34 | 1.96E-37 | 2.80E-54 | 2.46E-23 | 2.76E-06 | 1.74E-05 |
| IB-35 | 0.014917424 | 8.87E-19 | 2.00559E+14 | 0.823381181 | 0.871014804 |
| IB-36 | 8.76E-42 | 1.77E-68 | 6.00E-18 | 0.001333268 | 0.002820798 |
| IB-37 | 1.14E-09 | 2.54E-24 | 198331.3677 | 0.221697747 | 0.283773116 |
| IB-38 | 1.68E-44 | 4.23E-67 | 7.71E-25 | 4.41E-05 | 0.000152583 |
| IB-39 | 2.39E-09 | 2.84E-18 | 0.759356323 | 0.051015177 | 0.078674008 |
| IB-40 | 7.29E-20 | 4.57E-39 | 0.072493651 | 0.042287188 | 0.066414234 |
| IB-41 | 4.85E-57 | 5.87E-80 | 1.63E-38 | 7.45E-08 | 2.39E-06 |
| IB-42 | 6.03E-37 | 6.62E-57 | 1.49E-19 | 0.000130339 | 0.000374907 |
| IB-43 | 1.05E-13 | 1.20E-27 | 1.40875871 | 0.058490675 | 0.088080076 |
| IB-44 | 1.21E-23 | 1.69E-41 | 6.22E-07 | 0.008928525 | 0.015872934 |
| IB-45 | 1.04E-19 | 4.66E-35 | 2.16E-05 | 0.0115545 | 0.020259946 |
| IB-46 | 3.48E-36 | 7.40E-56 | 1.52E-18 | 0.000176458 | 0.000475508 |
| IB-47 | 4.72E-21 | 1.63E-32 | 1.11E-10 | 0.000237664 | 0.000620837 |
| IB-48 | 8.88E-70 | 1.35E-99 | 1.39E-46 | 2.42E-07 | 4.13E-06 |
| IB-49 | 1.03523E+11 | 1.35E-07 | 3.94E+29 | 0.233012156 | 0.295302535 |
| IB-50 | 1.85E-38 | 1.41E-57 | 5.50E-22 | 2.80E-05 | 0.000107126 |
| IB-51 | 2.44E-43 | 5.82E-68 | 1.01E-21 | 0.000273083 | 0.000685386 |
| IB-52 | 1.65E-21 | 7.44E-39 | 1.94E-05 | 0.014085865 | 0.024364739 |
| IB-53 | 2.10E-39 | 4.34E-67 | 4.94E-14 | 0.004008545 | 0.007715696 |
| IB-54 | 21.89895214 | 2.33E-16 | 2.7043E+18 | 0.876565816 | 0.912198573 |
| IB-55 | 6.17E-29 | 6.62E-50 | 1.35E-09 | 0.005701021 | 0.010575806 |
| IB-56 | 2.61E-52 | 1.54E-74 | 1.33E-33 | 6.40E-07 | 6.56E-06 |
| IB-57 | 9.26E-59 | 3.65E-84 | 1.79E-39 | 2.69E-07 | 4.30E-06 |
| IB-58 | 8.83E-30 | 1.49E-47 | 1.11E-14 | 0.000517192 | 0.001192804 |
| IB-59 | 8.10E-46 | 1.64E-67 | 1.94E-27 | 9.05E-06 | 4.37E-05 |
| IB-60 | 1.76E-52 | 2.71E-75 | 9.16E-34 | 8.29E-07 | 6.86E-06 |
| IB-61 | 3.25153E+18 | 359.9820936 | 2.60E+35 | 0.026274294 | 0.0431216 |
| IB-62 | 2.83E-49 | 7.57E-71 | 5.31E-32 | 7.47E-07 | 6.83E-06 |
| IB-63 | 9.82E-82 | 3.01E-114 | 2.77E-56 | 3.04E-08 | 2.37E-06 |
| IB-64 | 9.50125E+12 | 1.023794797 | 5.62E+26 | 0.055427943 | 0.084461627 |
| IB-65 | 2.86E-34 | 3.13E-51 | 2.81E-20 | 1.93E-05 | 8.26E-05 |
| IB-66 | 9.30E+46 | 3.97E+27 | 2.08E+69 | 8.61E-06 | 4.24E-05 |
| IB-67 | 4.16E-09 | 2.67E-30 | 1.83602E+12 | 0.429296944 | 0.501826564 |
| IB-68 | 7.320245082 | 6.76E-17 | 1.09907E+18 | 0.920443018 | 0.942533651 |
| IB-69 | 2.557605 | 2.44E-09 | 3400058865 | 0.929308223 | 0.944059147 |
| IB-70 | 6.21E-29 | 6.97E-46 | 7.98E-14 | 0.000509608 | 0.001185997 |
| IB-71 | 2.17E-32 | 2.73E-50 | 1.92E-16 | 0.000223284 | 0.000589287 |
| IB-72 | 4.12E-09 | 7.76E-25 | 8943613.978 | 0.287002521 | 0.356663326 |
| IB-73 | 5.15E-07 | 1.00E-14 | 12.80848908 | 0.100407605 | 0.138195413 |
| IB-74 | 2.28E-05 | 2.14E-16 | 1632468.191 | 0.402433496 | 0.479176628 |
| IB-75 | 1.05E-38 | 1.06E-58 | 1.41E-21 | 5.41E-05 | 0.000175218 |
| IB-76 | 1.04E-07 | 6.34E-16 | 7.786289913 | 0.086731637 | 0.124040778 |
| IB-77 | 1.393879193 | 3.67E-21 | 5.91E+20 | 0.988979664 | 0.989897571 |
| IB-78 | 2.12E-18 | 2.71E-40 | 1044.97677 | 0.101006125 | 0.138275765 |
| IB-79 | 1.28E-36 | 3.67E-55 | 1.88E-20 | 4.41E-05 | 0.000152583 |
| IB-80 | 0.000785573 | 4.74E-18 | 1.09721E+11 | 0.664908548 | 0.724325908 |
| IB-81 | 4.22E-31 | 4.55E-50 | 5.35E-14 | 0.000885397 | 0.00197097 |
| IB-82 | 7.11E-13 | 4.07E-25 | 0.160101579 | 0.041999911 | 0.06637023 |
| IB-83 | 4.49E-27 | 4.81E-44 | 1.01E-11 | 0.001292935 | 0.002781441 |
| IB-84 | 1.69E-17 | 3.14E-32 | 0.000576075 | 0.019162403 | 0.032487252 |
| IB-85 | 1.92E-14 | 1.55E-29 | 3.092024986 | 0.06466355 | 0.09680625 |
| IB-86 | 7.25E-06 | 9.69E-23 | 3.78333E+11 | 0.544872965 | 0.614482287 |
| IB-87 | 3.84E-34 | 2.56E-51 | 3.22E-19 | 3.93E-05 | 0.000139891 |
| IB-88 | 1.37E+21 | 13586381.21 | 3.57E+36 | 0.004540289 | 0.008673985 |
| IB-89 | 4.90E-96 | 4.40E-136 | 8.85E-66 | 7.21E-08 | 2.39E-06 |
| IB-90 | 1.88E-81 | 8.34E-117 | 1.89E-55 | 1.64E-07 | 3.22E-06 |
| IB-91 | 0.000294711 | 4.89E-09 | 9.189292769 | 0.132478835 | 0.178497799 |
| IB-92 | 1.05E-32 | 2.95E-49 | 4.80E-18 | 5.26E-05 | 0.000172667 |
| IB-93 | 4.21E-35 | 2.57E-53 | 4.59E-19 | 7.47E-05 | 0.000230371 |
| IB-94 | 3.49E-12 | 7.97E-22 | 0.002806996 | 0.014801531 | 0.025430819 |
| IB-95 | 7.22E-25 | 6.07E-40 | 4.30E-11 | 0.000961449 | 0.002103684 |
| IB-96 | 1.02954E+15 | 0.000349058 | 2.89E+34 | 0.117895802 | 0.160538965 |
| IB-97 | 0.039632775 | 2.48E-19 | 6.50737E+15 | 0.872244238 | 0.911406224 |
| IB-98 | 2.569949861 | 6.27E-17 | 1.33621E+17 | 0.961253309 | 0.968822232 |
| IB-99 | 1.80E-42 | 2.48E-61 | 1.36E-26 | 2.11E-06 | 1.38E-05 |
| IB-100 | 5.90E-90 | 1.05E-127 | 1.36E-58 | 3.32E-07 | 4.72E-06 |
| IB-101 | 9.11E-11 | 4.33E-23 | 38.16346148 | 0.097234718 | 0.135283087 |
| IB-102 | 1.48E-19 | 6.66E-35 | 3.24E-05 | 0.01233328 | 0.021478365 |
| IB-103 | 1.34E-31 | 1.07E-45 | 2.46E-19 | 3.82E-06 | 2.08E-05 |
| IB-104 | 2.13E-88 | 2.03E-125 | 5.68E-60 | 1.09E-07 | 2.55E-06 |
| IB-105 | 32.43404698 | 4.06E-31 | 3.10E+33 | 0.92559678 | 0.944034963 |
| IB-106 | 7.28E-41 | 1.49E-58 | 7.16E-26 | 1.24E-06 | 9.06E-06 |
| IB-107 | 2.21617E+11 | 0.000196562 | 9.12E+26 | 0.144538471 | 0.193186641 |
| IB-108 | 7.68E-33 | 6.55E-52 | 4.57E-16 | 0.000406653 | 0.001000991 |
| IB-109 | 2.33E-45 | 1.02E-63 | 2.53E-30 | 1.28E-07 | 2.73E-06 |
| IB-110 | 8.19E-61 | 1.05E-88 | 1.51E-37 | 3.47E-06 | 1.93E-05 |
| IB-111 | 0.000546813 | 2.83E-15 | 87536769.38 | 0.567305662 | 0.630360765 |
| IB-112 | 0.015477742 | 1.92E-10 | 1160748.719 | 0.650619954 | 0.711789352 |
| IB-113 | 0.811253126 | 6.64E-15 | 1.35726E+14 | 0.989897571 | 0.989897571 |
| IB-114 | 9.76981E+15 | 0.003351126 | 3.99E+35 | 0.096661624 | 0.135220632 |
| IB-115 | 1.37E-25 | 2.35E-40 | 2.29E-12 | 0.000453539 | 0.001075055 |
| IB-116 | 1.65E-96 | 2.89E-136 | 1.54E-66 | 4.63E-08 | 2.37E-06 |
| IB-117 | 9.60E-07 | 1.32E-29 | 4.3698E+16 | 0.601584432 | 0.660968303 |
| IB-118 | 1.74E-56 | 9.00E-81 | 1.25E-36 | 6.33E-07 | 6.56E-06 |
| IB-119 | 697493.0546 | 4.70E-09 | 2.22E+20 | 0.421173812 | 0.495142141 |
| IB-120 | 7.97E-20 | 1.87E-38 | 0.021284665 | 0.036585419 | 0.058173089 |
| IB-121 | 6.69E-27 | 1.20E-45 | 2.72E-10 | 0.003534943 | 0.006961119 |
| IB-122 | 84197.11042 | 6.12E-10 | 3.04E+19 | 0.498114388 | 0.569273587 |
| IB-123 | 3.96E-07 | 5.31E-23 | 1516244710 | 0.42164448 | 0.495142141 |
| IB-124 | 4.39E-16 | 4.30E-31 | 0.027806543 | 0.035246488 | 0.056394381 |
| IB-125 | 2.60E-49 | 2.59E-72 | 3.44E-30 | 5.21E-06 | 2.72E-05 |
| IB-126 | 1.85E-43 | 1.65E-65 | 6.61E-24 | 5.08E-05 | 0.000168755 |
| IB-127 | 5.51E-07 | 2.56E-23 | 6077455343 | 0.445703041 | 0.518636266 |
| IB-128 | 1.07E-64 | 3.65E-97 | 8.91E-38 | 2.04E-05 | 8.42E-05 |
| IB-129 | 2.57E-42 | 5.04E-62 | 1.98E-25 | 7.38E-06 | 3.78E-05 |
| IB-130 | 1.10E-06 | 3.48E-15 | 170.6460267 | 0.158772067 | 0.208439226 |
| IB-131 | 7.50E-29 | 4.96E-46 | 9.63E-14 | 0.000586675 | 0.001338411 |
| IB-132 | 4.12E+73 | 8.99E+44 | 1.32E+107 | 2.94E-06 | 1.79E-05 |
| IB-133 | 4.38E-27 | 7.03E-40 | 5.28E-16 | 1.31E-05 | 6.01E-05 |
| IB-134 | 6.22E-15 | 2.69E-31 | 20.73996599 | 0.078717804 | 0.114498624 |
| IB-135 | 5.12E-19 | 1.84E-39 | 3.86337267 | 0.066345879 | 0.098747355 |
| IB-136 | 1.48E-16 | 2.25E-28 | 1.43E-05 | 0.00617008 | 0.011363601 |
| IB-137 | 3.44E-30 | 2.21E-46 | 2.64E-16 | 0.000108965 | 0.00031699 |
| IB-138 | 2.40E-19 | 1.34E-31 | 2.89E-08 | 0.001644375 | 0.003367679 |
| IB-139 | 7.44E-60 | 6.02E-85 | 9.52E-40 | 2.28E-07 | 4.13E-06 |
| IB-140 | 7638.147492 | 8.13E-20 | 1.41E+27 | 0.74025496 | 0.802988431 |
| IB-141 | 1.07E-24 | 1.77E-37 | 1.79E-13 | 8.09E-05 | 0.00024082 |
| IB-142 | 0.034793527 | 6.21E-24 | 1.31E+20 | 0.894307476 | 0.923156104 |
| IB-143 | 3.75E-09 | 1.22E-32 | 4.47057E+14 | 0.475281709 | 0.548072601 |
| IB-144 | 2.02E-20 | 2.44E-40 | 0.068854794 | 0.043151227 | 0.067358013 |
| IB-145 | 1.15E-58 | 2.18E-84 | 3.03E-38 | 6.97E-07 | 6.63E-06 |
| IB-146 | 1.72E-37 | 2.48E-54 | 3.84E-23 | 3.34E-06 | 1.90E-05 |
| IB-147 | 1.13E-25 | 6.26E-40 | 6.27E-13 | 0.000267213 | 0.000677293 |
| IB-148 | 3.81E-19 | 6.76E-36 | 0.000896087 | 0.023560972 | 0.039681638 |
| IB-149 | 7.57E-19 | 8.88E-32 | 4.07E-07 | 0.003740671 | 0.007254634 |
| IB-150 | 1.97E-23 | 4.67E-35 | 2.84E-13 | 3.84E-05 | 0.000138607 |
| IB-151 | 2.79E-38 | 7.60E-65 | 6.25E-14 | 0.003579367 | 0.006994794 |
| IB-152 | 1.73E-41 | 3.95E-63 | 4.29E-23 | 6.12E-05 | 0.000193301 |
| IB-153 | 8.90E-14 | 1.83E-26 | 0.027487481 | 0.03249059 | 0.052311894 |
| IB-154 | 3.92E-50 | 6.95E-73 | 2.75E-31 | 2.79E-06 | 1.74E-05 |
| IB-155 | 3.29E-43 | 1.16E-64 | 4.79E-25 | 2.24E-05 | 9.02E-05 |
| IB-156 | 2.91E-20 | 4.71E-30 | 1.33E-11 | 2.92E-05 | 0.000109915 |
| IB-157 | 8.84E-48 | 6.21E-74 | 1.68E-24 | 0.00017111 | 0.00047531 |
| IB-158 | 5.35E-51 | 1.50E-71 | 3.45E-34 | 1.02E-07 | 2.55E-06 |
| IB-159 | 1.70E-20 | 1.82E-36 | 7.02E-06 | 0.010697526 | 0.018886667 |
| IB-160 | 5.15E-40 | 4.09E-62 | 1.50E-20 | 0.000187482 | 0.000499953 |
| IB-161 | 0.357672767 | 0.000394138 | 253.4625763 | 0.760671597 | 0.814777945 |
| IB-162 | 1.53E-10 | 6.01E-22 | 11.78935558 | 0.08228419 | 0.119009902 |
| IB-163 | 4.27E+151 | 6.10E+101 | 3.47E+214 | 1.08E-07 | 2.55E-06 |
| IB-164 | 5.27E-05 | 4.91E-12 | 355.6538651 | 0.223027208 | 0.284054554 |
| IB-165 | 94.71010627 | 3.54E-22 | 4.45E+25 | 0.868290192 | 0.910992989 |
| IB-166 | 3.09E+28 | 1.71393E+15 | 3.88E+43 | 7.19E-05 | 0.000224565 |
| IB-167 | 2.57E-05 | 3.41E-21 | 76284686706 | 0.562356436 | 0.628660469 |
| IB-168 | 4.07E-31 | 2.22E-54 | 2.47E-10 | 0.006496268 | 0.011878889 |
| IB-169 | 1.517E+11 | 2.14E-08 | 9.79E+30 | 0.252115104 | 0.316379739 |
| IB-170 | 3.11E-06 | 6.55E-13 | 4.448900941 | 0.089591994 | 0.127419725 |
| IB-171 | 5.26E-13 | 2.74E-22 | 0.000267991 | 0.007104342 | 0.012898663 |
| IB-172 | 27031109.6 | 1.20E-08 | 1.40E+23 | 0.344743594 | 0.416294152 |
| IB-173 | 9.16E-27 | 1.55E-40 | 1.47E-14 | 7.61E-05 | 0.00023193 |
| IB-174 | 1.94E-06 | 1.48E-14 | 125.3638452 | 0.157120916 | 0.207334817 |
| IB-175 | 0.000857532 | 5.43E-14 | 9327895.408 | 0.549454075 | 0.616930891 |
| IB-176 | 7.49E-36 | 2.74E-53 | 5.42E-21 | 1.76E-05 | 7.62E-05 |
| IB-177 | 2.15E-09 | 1.41E-30 | 9.45695E+11 | 0.413219286 | 0.489741376 |
| IB-178 | 9.728013101 | 2.18E-13 | 5.19637E+14 | 0.886707216 | 0.919016386 |
| IB-179 | 1.55E-13 | 2.58E-24 | 0.001728883 | 0.015155896 | 0.025866063 |
| IB-180 | 9.70E-23 | 2.57E-35 | 1.44E-11 | 0.000256705 | 0.000663803 |
| IB-181 | 6.37E-19 | 1.30E-31 | 4.16E-07 | 0.003409717 | 0.006769584 |
| IB-182 | 3.39E-79 | 1.91E-114 | 2.04E-50 | 1.28E-06 | 9.14E-06 |
| IB-183 | 746360703 | 4.67E-07 | 4.33E+24 | 0.257221213 | 0.321212832 |
| IB-184 | 6.98E-25 | 2.24E-37 | 7.00E-14 | 4.99E-05 | 0.000168068 |
| IB-185 | 2.59E-06 | 1.69E-17 | 218898.1073 | 0.318583906 | 0.390227176 |
| IB-186 | 1.30E-32 | 2.97E-46 | 5.39E-21 | 6.25E-07 | 6.56E-06 |
| IB-187 | 2.95E-23 | 6.84E-36 | 3.19E-12 | 0.000175186 | 0.000475508 |
| IB-188 | 1.35E-20 | 6.42E-39 | 0.001259783 | 0.026218035 | 0.0431216 |
| IB-189 | 1.58E-32 | 2.31E-51 | 1.15E-15 | 0.000436565 | 0.001064386 |
| IB-190 | 8.08E-07 | 1.85E-16 | 1879.975039 | 0.205885156 | 0.267546193 |
| IB-191 | 1.13659E+18 | 0.05252598 | 2.96E+38 | 0.072938658 | 0.107312048 |
| IB-192 | 1.27E-61 | 6.12E-88 | 1.29E-39 | 6.41E-07 | 6.56E-06 |
| IB-193 | 1.94E-52 | 2.69E-77 | 1.21E-31 | 7.60E-06 | 3.82E-05 |
| IB-194 | 2.00E-22 | 4.08E-36 | 3.24E-10 | 0.00092509 | 0.002041578 |
| IB-195 | 7.32E-12 | 3.16E-22 | 0.051071229 | 0.029771354 | 0.04823713 |
| IB-196 | 3.16E-05 | 1.53E-12 | 303.8176807 | 0.213797988 | 0.276425681 |
| IB-197 | 4.62E-07 | 2.83E-17 | 4570.242631 | 0.216272267 | 0.2782196 |
| IB-198 | 7.98E-13 | 3.58E-40 | 3.7686E+14 | 0.376093547 | 0.449906299 |
| IB-199 | 9.65E-10 | 6.46E-27 | 63075787.22 | 0.29539641 | 0.363564812 |
| IB-200 | 1.83E-12 | 2.01E-26 | 24.13001669 | 0.086630603 | 0.124040778 |
| IB-201 | 3.81E-19 | 1.19E-38 | 1.073832765 | 0.055234263 | 0.084461627 |
| IB-202 | 1.23E-74 | 5.99E-108 | 1.13E-46 | 1.93E-06 | 1.30E-05 |
| IB-203 | 7.66E-14 | 2.43E-32 | 23888.01028 | 0.14826556 | 0.196663127 |
| IB-204 | 1.53E-64 | 4.66E-90 | 5.50E-44 | 4.26E-08 | 2.37E-06 |
| IB-205 | 3.45E-67 | 1.52E-97 | 2.02E-41 | 3.03E-06 | 1.80E-05 |
| IB-206 | 7.29345E+11 | 8.26E-07 | 3.16E+30 | 0.200133415 | 0.261398746 |
| IB-207 | 1.44E-32 | 8.30E-53 | 4.37E-14 | 0.001182146 | 0.002564656 |
| IB-208 | 2.86E-22 | 1.35E-34 | 2.71E-11 | 0.000265201 | 0.000677293 |
| IB-209 | 1.32E-48 | 7.27E-70 | 3.00E-31 | 9.23E-07 | 7.16E-06 |
| IB-210 | 1.20E-16 | 2.60E-28 | 8.82E-06 | 0.005309565 | 0.010029638 |
| IB-211 | 1.74E-43 | 1.34E-61 | 1.48E-28 | 3.05E-07 | 4.59E-06 |
| IB-212 | 3.48E-52 | 8.54E-75 | 1.39E-33 | 8.18E-07 | 6.86E-06 |
| IB-213 | 2.66E-12 | 1.81E-32 | 45283432.46 | 0.245734415 | 0.309891676 |
| IB-214 | 6.27E-19 | 6.27E-33 | 5.23E-06 | 0.007611952 | 0.013722956 |
| IB-215 | 0.001965731 | 8.87E-21 | 3.4531E+14 | 0.756993183 | 0.814244768 |
| IB-216 | 0.005450792 | 2.55E-19 | 1.0008E+14 | 0.784021984 | 0.832820033 |
| IB-217 | 5.54E-53 | 1.07E-75 | 3.38E-34 | 6.99E-07 | 6.63E-06 |
| IB-218 | 2.12E-23 | 1.73E-36 | 8.95E-12 | 0.000293326 | 0.000729042 |
| IB-219 | 1.46E-35 | 1.41E-56 | 1.47E-16 | 0.000590783 | 0.001338411 |
| IB-220 | 1.73E-71 | 6.63E-103 | 6.51E-47 | 5.85E-07 | 6.56E-06 |
| IB-221 | 1.05E-59 | 1.51E-85 | 1.35E-39 | 3.68E-07 | 4.96E-06 |
| IB-222 | 0.002037458 | 5.10E-13 | 7846920.52 | 0.579859934 | 0.639845444 |
| IB-223 | 1.68E-07 | 4.45E-31 | 3.48545E+16 | 0.568802096 | 0.630360765 |
| IB-224 | 1.26E-05 | 7.96E-19 | 132264556 | 0.460769999 | 0.533742623 |
| IB-225 | 2.07E-35 | 2.76E-50 | 6.49E-23 | 5.66E-07 | 6.56E-06 |
| IB-226 | 2.88E-36 | 2.25E-53 | 1.31E-21 | 1.03E-05 | 4.90E-05 |
| IB-227 | 0.000106831 | 4.73E-13 | 16667.63518 | 0.343392218 | 0.416294152 |
| IB-228 | 1.11E-07 | 4.40E-30 | 1.12343E+15 | 0.536009552 | 0.607161262 |
| IB-229 | 4.12E-17 | 9.88E-36 | 16.737159 | 0.074116298 | 0.108421556 |
| IB-230 | 9.59E-24 | 8.39E-39 | 1.98E-10 | 0.001329067 | 0.002820798 |
| IB-231 | 1.04E-15 | 1.58E-32 | 10.24368902 | 0.071836957 | 0.106302087 |
| IB-232 | 1.54E-32 | 3.56E-47 | 6.48E-20 | 4.09E-06 | 2.18E-05 |
| IB-233 | 1.07E-25 | 1.10E-40 | 1.54E-12 | 0.000475909 | 0.001117732 |
| IB-234 | 1.53E-21 | 3.60E-35 | 2.07E-09 | 0.001415623 | 0.002946338 |
| IB-235 | 1.46E-13 | 2.16E-25 | 0.013066683 | 0.026277225 | 0.0431216 |
| IB-236 | 278380556.6 | 2.14E-16 | 7.64E+32 | 0.492485379 | 0.565364382 |
| IB-237 | 3.33E-38 | 5.20E-57 | 3.00E-22 | 2.26E-05 | 9.02E-05 |
| IB-238 | 5.74E-31 | 9.11E-46 | 3.24E-18 | 1.46E-05 | 6.45E-05 |
| IB-239 | 56010.56355 | 1.83E-24 | 3.36E+33 | 0.743458362 | 0.80306051 |
| IB-240 | 2.26E-38 | 2.37E-57 | 3.52E-22 | 2.37E-05 | 9.31E-05 |
| IB-241 | 8.77E-58 | 3.37E-87 | 4.40E-33 | 3.19E-05 | 0.000118432 |
| IB-242 | 7.20E-67 | 5.57E-93 | 3.90E-46 | 2.24E-08 | 2.37E-06 |
| IB-243 | 4.04E-22 | 7.39E-34 | 1.07E-11 | 0.000138896 | 0.000395083 |
| IB-244 | 7.13E-15 | 5.68E-30 | 1.610534877 | 0.057868882 | 0.087659371 |
| IB-245 | 3.48E-17 | 6.16E-40 | 225219.4071 | 0.14488998 | 0.193186641 |
| IB-246 | 5.41E-06 | 5.58E-16 | 31711.42952 | 0.292658247 | 0.361934837 |
| IB-247 | 7.78E-49 | 8.61E-73 | 3.04E-28 | 2.03E-05 | 8.42E-05 |
| IB-248 | 3.55897E+18 | 5413396.828 | 5.74E+31 | 0.003411235 | 0.006769584 |
| IB-249 | 2.76E-16 | 2.27E-35 | 313.6230808 | 0.098235664 | 0.135936919 |
| IB-250 | 5.89E-88 | 1.86E-122 | 1.15E-59 | 4.08E-08 | 2.37E-06 |
| IB-251 | 2.40E-36 | 2.06E-55 | 8.29E-20 | 7.89E-05 | 0.000237772 |
| IB-252 | 7.76E-08 | 2.50E-16 | 9.931775004 | 0.091058959 | 0.128790572 |
| IB-253 | 5.34E-68 | 6.12E-99 | 5.75E-42 | 3.15E-06 | 1.83E-05 |
| IB-254 | 3.70E-11 | 1.39E-33 | 2.42668E+11 | 0.352312468 | 0.423436581 |
| IB-255 | 3.66E-40 | 1.22E-63 | 2.35E-19 | 0.000448962 | 0.001074152 |
| IB-256 | 2.47E-56 | 3.63E-81 | 5.15E-36 | 1.10E-06 | 8.31E-06 |
| Abbreviation: IB, Imaging biomarker; OR, Odds ratio; FDR, False discovery rate; CI, Confidence interval. | | | | | |
