# Supplementary material for: Development and validation of chest CT-based imaging biomarkers for early stage COVID-19 screening
Source: Front Public Health. 2022 Sep 21;10:1004117. doi: 10.3389/fpubh.2022.1004117 (PMC9533142; doi:10.3389/fpubh.2022.1004117)
Supplement: Supplementary file 4 [file Table_4.docx]

**Supplementary Table 4** COVID-19 screening performance on the combination of both training and validation cohorts.

| Feature | AUC (95%CI) | Sensitivity (95%CI) | Specificity (95%CI) | Accuracy (95%CI) | F1 score (95%CI) | Precision (95%CI) |
| --- | --- | --- | --- | --- | --- | --- |
| Signal | 0.824 (0.745, 0.880) | 0.841 (0.614, 1.000) | 0.881 (0.575, 1.000) | 0.857 (0.639, 0.988) | 0.857 (0.651, 0.987) | 0.876 (0.643, 1.000) |
| IB-3 | 0.850 (0.771, 0.935) | 0.750 (0.650, 0.900) | 0.818 (0.750, 0.921) | 0.793 (0.732, 0.856) | 0.775 (0.690, 0.849) | 0.795 (0.732, 0.898) |
| IB-61 | 0.890 (0.831, 0.940) | 0.787 (0.675, 0.888) | 0.795 (0.659, 0.898) | 0.791 (0.721, 0.861) | 0.782 (0.715, 0.852) | 0.778 (0.688, 0.881) |
| IB-66 | 0.883 (0.824, 0.933) | 0.775 (0.650, 0.875) | 0.795 (0.693, 0.886) | 0.775 (0.714, 0.855) | 0.771 (0.697, 0.845) | 0.770 (0.690, 0.867) |
| IB-88 | 0.832 (0.748, 0.905) | 0.725 (0.600, 0.875) | 0.773 (0.614, 0.887) | 0.747 (0.663, 0.830) | 0.732 (0.637, 0.829) | 0.743 (0.641, 0.859) |
| IB-132 | 0.892 (0.839, 0.945) | 0.825 (0.700, 0.900) | 0.818 (0.693, 0.921) | 0.809 (0.742, 0.899) | 0.802 (0.724, 0.893) | 0.791 (0.710, 0.906) |
| IB-163 | 0.900 (0.842, 0.953) | 0.825 (0.675, 0.900) | 0.841 (0.715, 0.944) | 0.824 (0.760, 0.893) | 0.819 (0.742, 0.889) | 0.821 (0.730, 0.926) |
| IB-166 | 0.887 (0.829, 0.935) | 0.800 (0.675, 0.900) | 0.795 (0.659, 0.932) | 0.795 (0.707, 0.859) | 0.788 (0.698, 0.849) | 0.780 (0.682, 0.903) |
| IB-248 | 0.860 (0.808, 0.934) | 0.750 (0.637, 0.850) | 0.818 (0.705, 0.921) | 0.784 (0.701, 0.843) | 0.769 (0.680, 0.836) | 0.782 (0.695, 0.893) |
| Imaging biomarkers (All) | 0.980 (0.937, 0.999) | 0.950 (0.850, 1.000) | 0.977 (0.909, 1.000) | 0.953 (0.909, 0.995) | 0.951 (0.903, 0.994) | 0.973 (0.902, 1.000) |
