# Supplementary material for: Development and validation of chest CT-based imaging biomarkers for early stage COVID-19 screening
Source: Front Public Health. 2022 Sep 21;10:1004117. doi: 10.3389/fpubh.2022.1004117 (PMC9533142; doi:10.3389/fpubh.2022.1004117)
Supplement: Supplementary file 5 [file Table_5.docx]

**Supplementary Table 5** Characteristics of study population stratified by age groups

| Variable | All | | | Training set | | | Validation set | | |
| --- | --- | --- | --- | --- | --- | --- | --- | --- | --- |
|  | COVID-19 | non-COVID-19 | P value | COVID-19 | non-COVID-19 | P value | COVID-19 | non-COVID-19 | P value |
|  | (n=200) | (n=219) |  | (n=47) | (n=69) |  | (n=153) | (n=150) |  |
| **All** |  |  |  |  |  |  |  |  |  |
| Age~ Median [95%CI ] | 61.0 [50.0, 68.2] | 37.5 [29.0, 49.0] | <0.001 | 53.0 [48.0, 61.0] | 34.5 [26.0, 42.2] | <0.001 | 64.0 [51.0, 70.0] | 39.0 [31.0, 51.0] | <0.001 |
| Gender |  |  |  |  |  |  |  |  |  |
| Female | 105 (52.5%) | 109 (49.8%) | 0.645 | 24 (51.1%) | 29 (42.0%) | 0.442 | 81 (52.9%) | 80 (53.3%) | 1 |
| Male | 95 (47.5%) | 110 (50.2%) |  | 23 (48.9%) | 40 (58.0%) |  | 72 (47.1%) | 70 (46.7%) |  |
| **Age<60 (n=287)** |  |  |  |  |  |  |  |  |  |
| Age~ Median [95%CI ] | 50.0 [43.0, 55.0] | 35.0 [27.0, 43.0] | <0.001 | 51.0 [44.5, 54.0] | 34.0 [26.0, 40.0] | <0.001 | 49.0 [43.0, 55.0] | 36.0 [29.0, 45.5] | <0.001 |
| Gender |  |  |  |  |  |  |  |  |  |
| Female | 53 (55.8%) | 98 (51.0%) | 0.527 | 20 (57.1%) | 27 (41.5%) | 0.2 | 33 (55.0%) | 71 (55.9%) | 1 |
| Male | 42 (44.2%) | 94 (49.0%) |  | 15 (42.9%) | 38 (58.5%) |  | 27 (45.0%) | 56 (44.1%) |  |
| **Age≥60 (n=131)** |  |  |  |  |  |  |  |  |  |
| Age~ Median [95%CI ] | 68.0 [65.0, 74.0] | 67.5 [63.0, 71.8] | 0.358 | 67.5 [64.8, 72.2] | 70.0 [67.5, 73.0] | 0.426 | 68.0 [65.0, 74.0] | 67.0 [62.5, 71.5] | 0.261 |
| Gender |  |  |  |  |  |  |  |  |  |
| Female | 52 (49.5%) | 10 (38.5%) | 0.428 | 4 (33.3%) | 1 (33.3%) | 1 | 48 (51.6%) | 9 (39.1%) | 0.401 |
| Male | 53 (50.5%) | 16 (61.5%) |  | 8 (66.7%) | 2 (66.7%) |  | 45 (48.4%) | 14 (60.9%) |  |
