# Supplementary material for: Development and validation of chest CT-based imaging biomarkers for early stage COVID-19 screening
Source: Front Public Health. 2022 Sep 21;10:1004117. doi: 10.3389/fpubh.2022.1004117 (PMC9533142; doi:10.3389/fpubh.2022.1004117)
Supplement: Supplementary file 6 [file Table_6.docx]

| **Supplementary Table 6** Spearman correlation of vasculature-like signal and individual imaging biomarker on combined cohorts | | | | | | |
| --- | --- | --- | --- | --- | --- | --- |
| Feature | COVID-19 | | | NON-COVID-19 | |  |
|  | Age correlation | P Value | FDR Adjusted P Value | Age correlation | P Value | FDR Adjusted P Value |
| Signal | 0.130642137 | 0.06519937 | 0.19559811 | -0.128277123 | 0.058635066 | 0.0879526 |
| IB-3 | -0.187893929 | 0.007713997 | 0.06942597 | -0.20279484 | 0.002626815 | 0.01118459 |
| IB-61 | -0.093618756 | 0.187313508 | 0.28097026 | -0.195655085 | 0.003728197 | 0.01118459 |
| IB-66 | -0.107775601 | 0.128749506 | 0.23174911 | -0.1318962 | 0.051807172 | 0.0879526 |
| IB-88 | -0.078004863 | 0.272241816 | 0.30627204 | 0.116012995 | 0.087480895 | 0.11247544 |
| IB-132 | -0.078173083 | 0.271206017 | 0.30627204 | -0.143976426 | 0.033616064 | 0.07563614 |
| IB-163 | -0.168798243 | 0.016877037 | 0.07594667 | -0.074156209 | 0.275664599 | 0.2756646 |
| IB-166 | -0.072195786 | 0.309663066 | 0.30966307 | -0.214293725 | 0.001458203 | 0.01118459 |
| IB-248 | 0.113411201 | 0.109823587 | 0.23174911 | 0.096368976 | 0.156192793 | 0.17571689 |
| Abbreviation: IB, Imaging biomarker; FDR, False discovery rate | | | |  |  |  |
