# Supplementary material for: Development and validation of chest CT-based imaging biomarkers for early stage COVID-19 screening
Source: Front Public Health. 2022 Sep 21;10:1004117. doi: 10.3389/fpubh.2022.1004117 (PMC9533142; doi:10.3389/fpubh.2022.1004117)
Supplement: Supplementary file 7 [file Table_7.docx]

**Supplementary Table 7**. Performance comparison of prediction models on the training set with cross validation (100 bootstrap iterations, 80% training sample rate) based on significant imaging biomarkers and biomarkers combined with age.

| Performance | Biomarkers only | Biomarkers combined with age | p value |
| --- | --- | --- | --- |
| Sensitivity [95%CI] | 1.00 [1.00, 1.00] | 1.00 [1.00, 1.00] | 0.207 |
| Specificity [95%CI] | 1.00 [0.93, 1.00] | 1.00 [0.93, 1.00] | 0.267 |
| AUC [95%CI] | 1.00 [1.00, 1.00] | 1.00 [0.99, 1.00] | 0.080 |
| Precision [95%CI] | 1.00 [0.93, 1.00] | 1.00 [0.93, 1.00] | 0.221 |
| Recall [95%CI] | 1.00 [1.00, 1.00] | 1.00 [1.00, 1.00] | 0.171 |
