# Supplementary material for: Development and validation of chest CT-based imaging biomarkers for early stage COVID-19 screening
Source: Front Public Health. 2022 Sep 21;10:1004117. doi: 10.3389/fpubh.2022.1004117 (PMC9533142; doi:10.3389/fpubh.2022.1004117)
Supplement: Supplementary file 8 [file Table_8.docx]

**Supplementary Table 8**. Performance comparison of prediction models on the validation cohort based on significant imaging biomarkers and biomarkers combined with age.

| Features | AUC | Sensitivity | Specificity | Recall | Precision |
| --- | --- | --- | --- | --- | --- |
| Biomarkers only | 0.971 | 0.941 | 0.92 | 0.92 | 0.939 |
| Biomarkers combined with age | 0.966 | 0.876 | 0.96 | 0.96 | 0.889 |
