# Supplementary material for: Development and validation of chest CT-based imaging biomarkers for early stage COVID-19 screening
Source: Front Public Health. 2022 Sep 21;10:1004117. doi: 10.3389/fpubh.2022.1004117 (PMC9533142; doi:10.3389/fpubh.2022.1004117)
Supplement: Supplementary file 22 [file Data_Sheet_1.pdf]

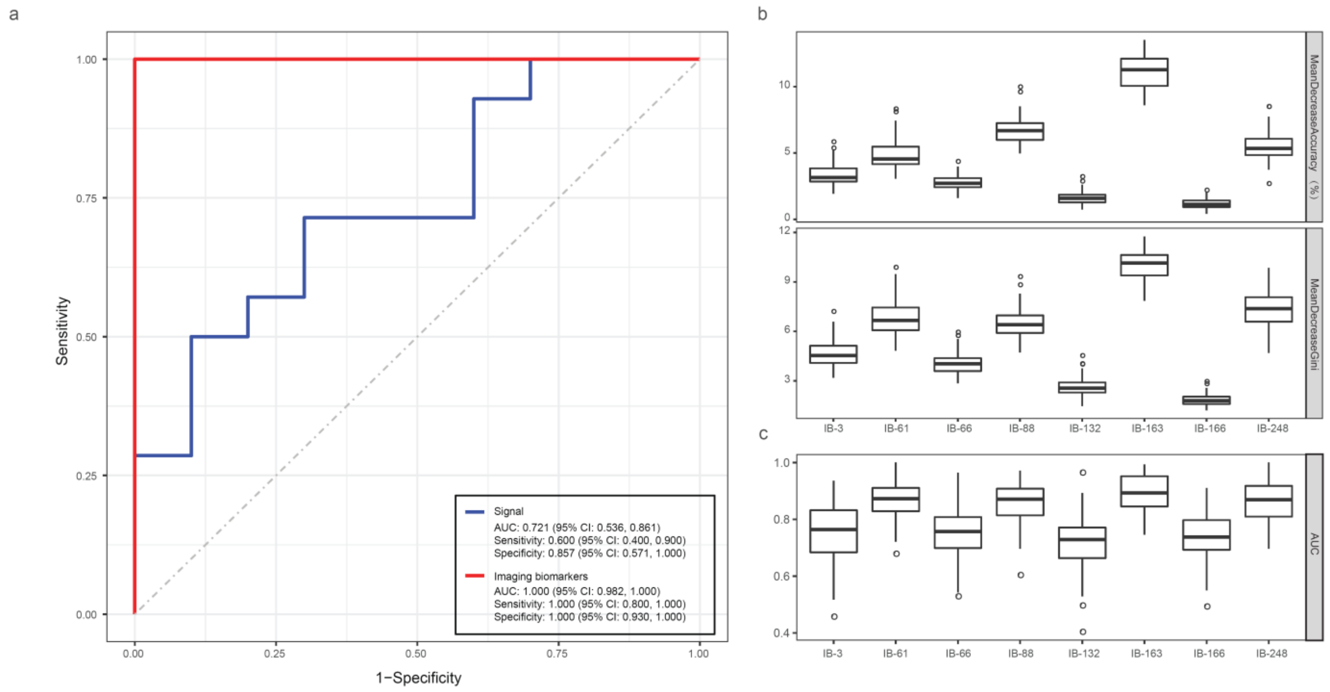

Supplementary Fig. 1 a. COVID-19 screening performance of signal-based model (blue line) and imaging biomarker-based model (red line) on training cohort. **b**. The importance of individual imaging biomarker during COVID-19 screening on training cohort assessed by random forest. **c**. The performance (AUC) of individual imaging biomarkers for COVID-19 screening on training cohort.

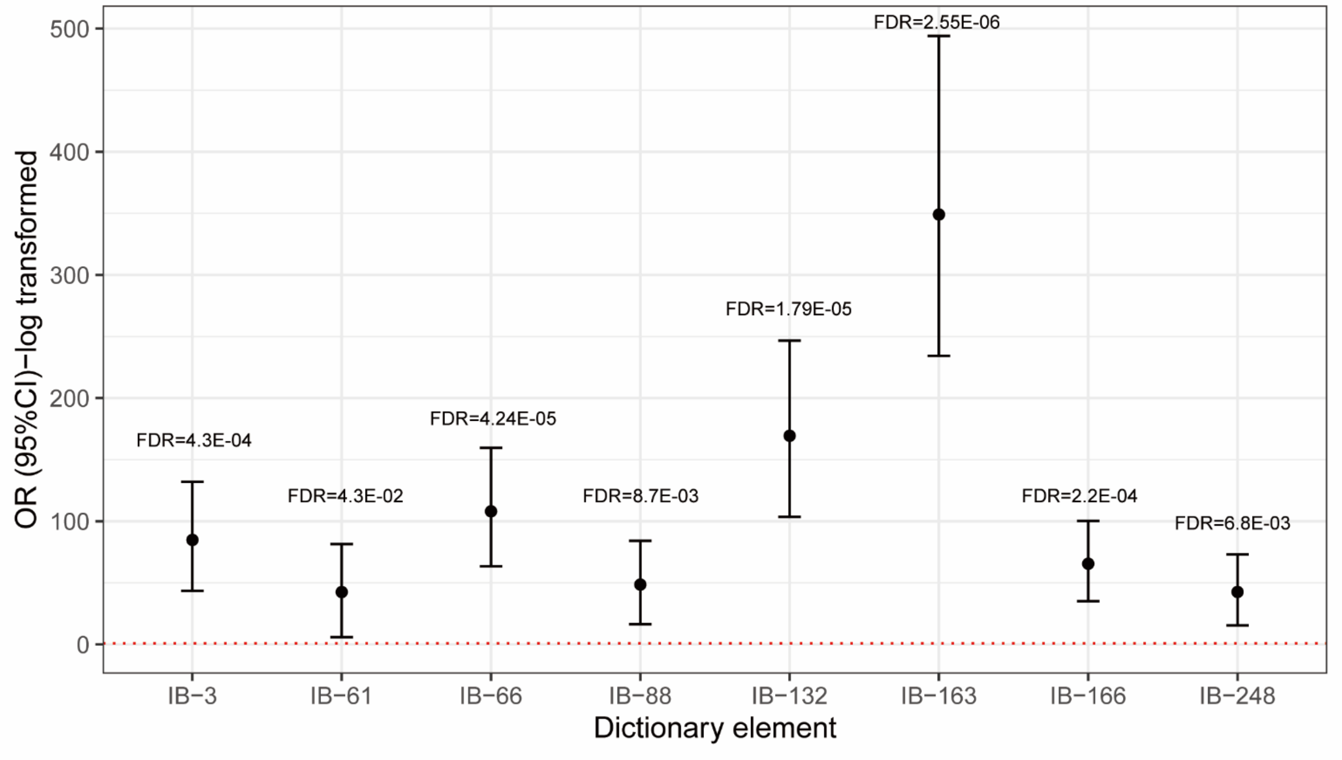

Supplementary Fig. 2 Significant dictionary elements (imaging biomarkers) positively correlated with COVID-19.

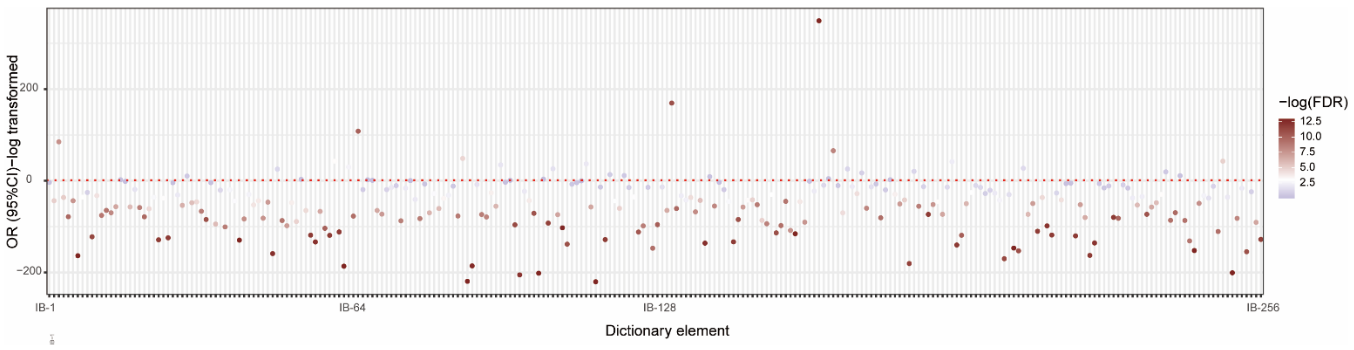

Supplementary Fig. 3 Associations between dictionary elements and COVID-19.

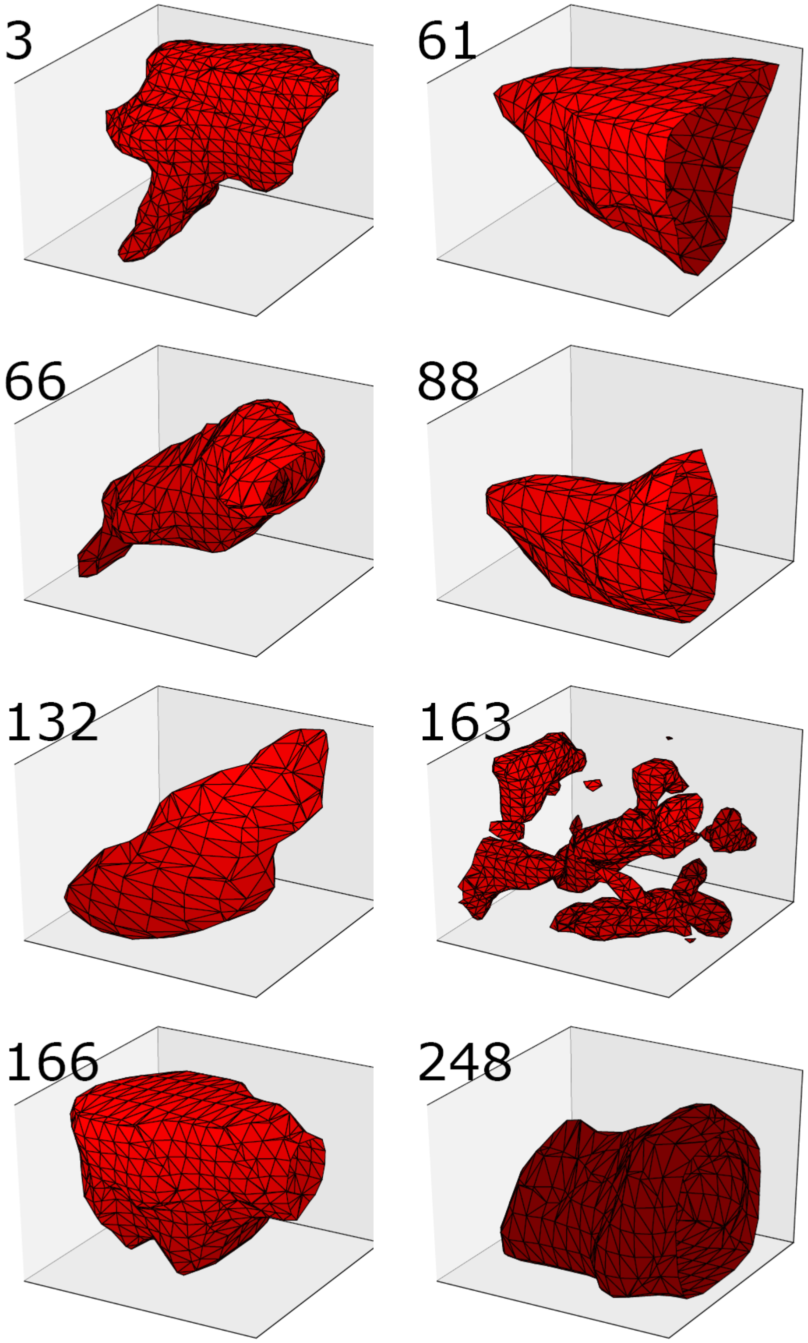

Supplementary Fig. 4 Visualization of 8 dictionary elements (i.e., Imaging Biomarkers) learned from training cohort that have significant positive correlation with COVID-19.

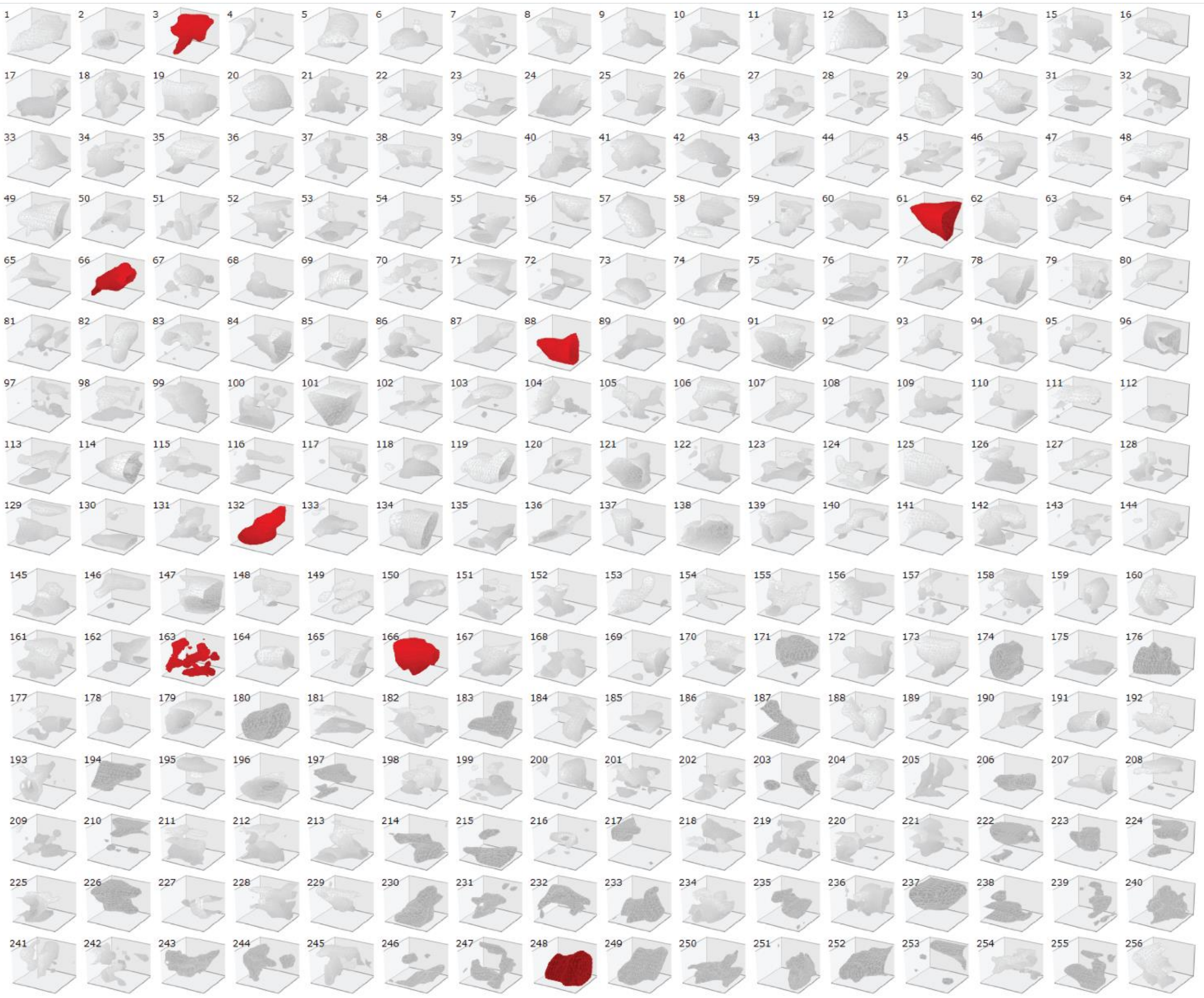

Supplementary Fig. 5 Visualization of 256 dictionary elements (i.e., signal patterns) learned from training cohort, where 8 (in red) dictionary elements have significant positive correlation with COVID-19. These 8 dictionary elements, served as our imaging biomarkers, have also been demonstrated in Figure 1 and Supplementary Figure 2.

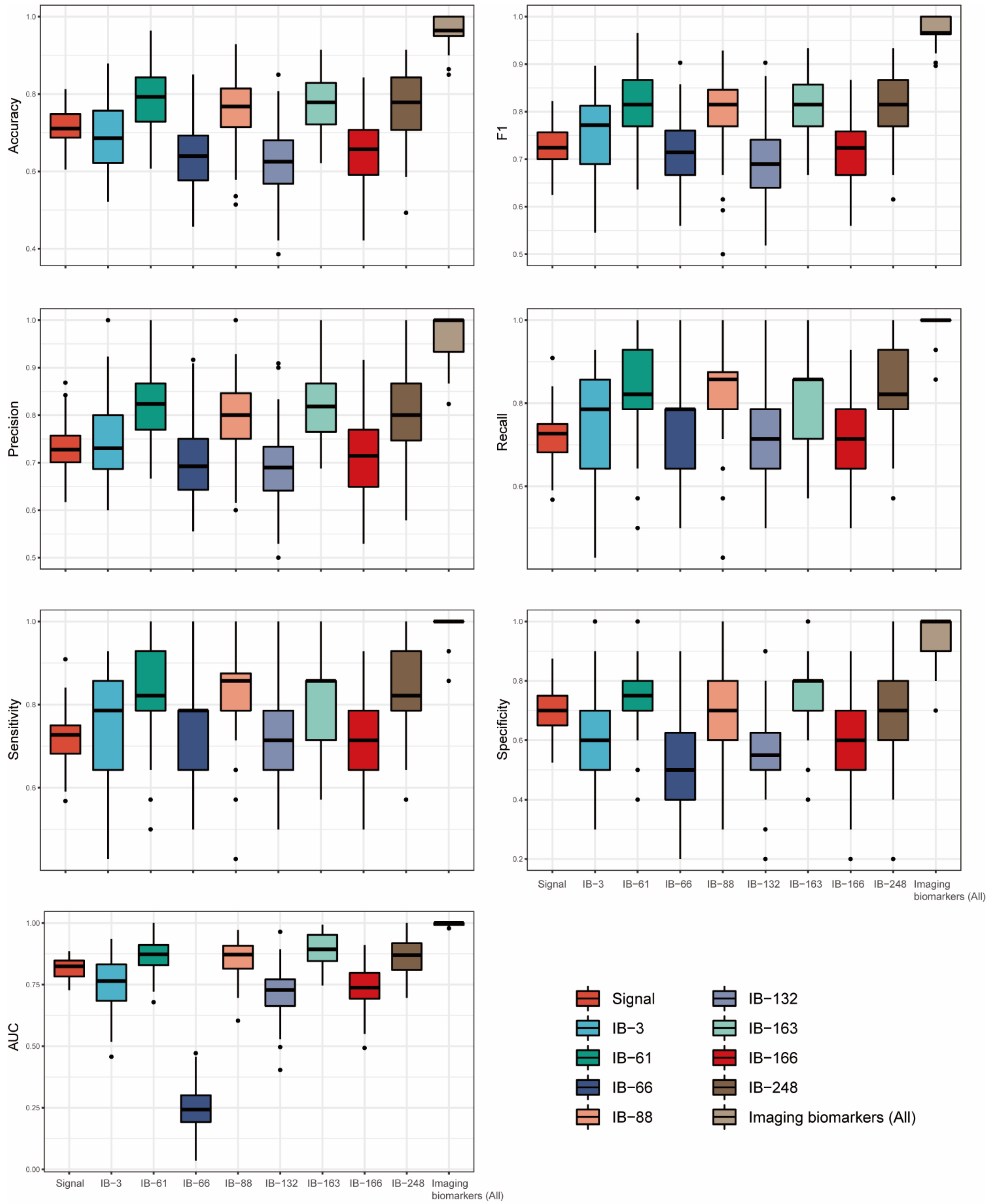

Supplementary Fig. 6 COVID-19 screening performance on training cohort with vasculature-like signal, individual imaging biomarker and all imaging biomarkers.

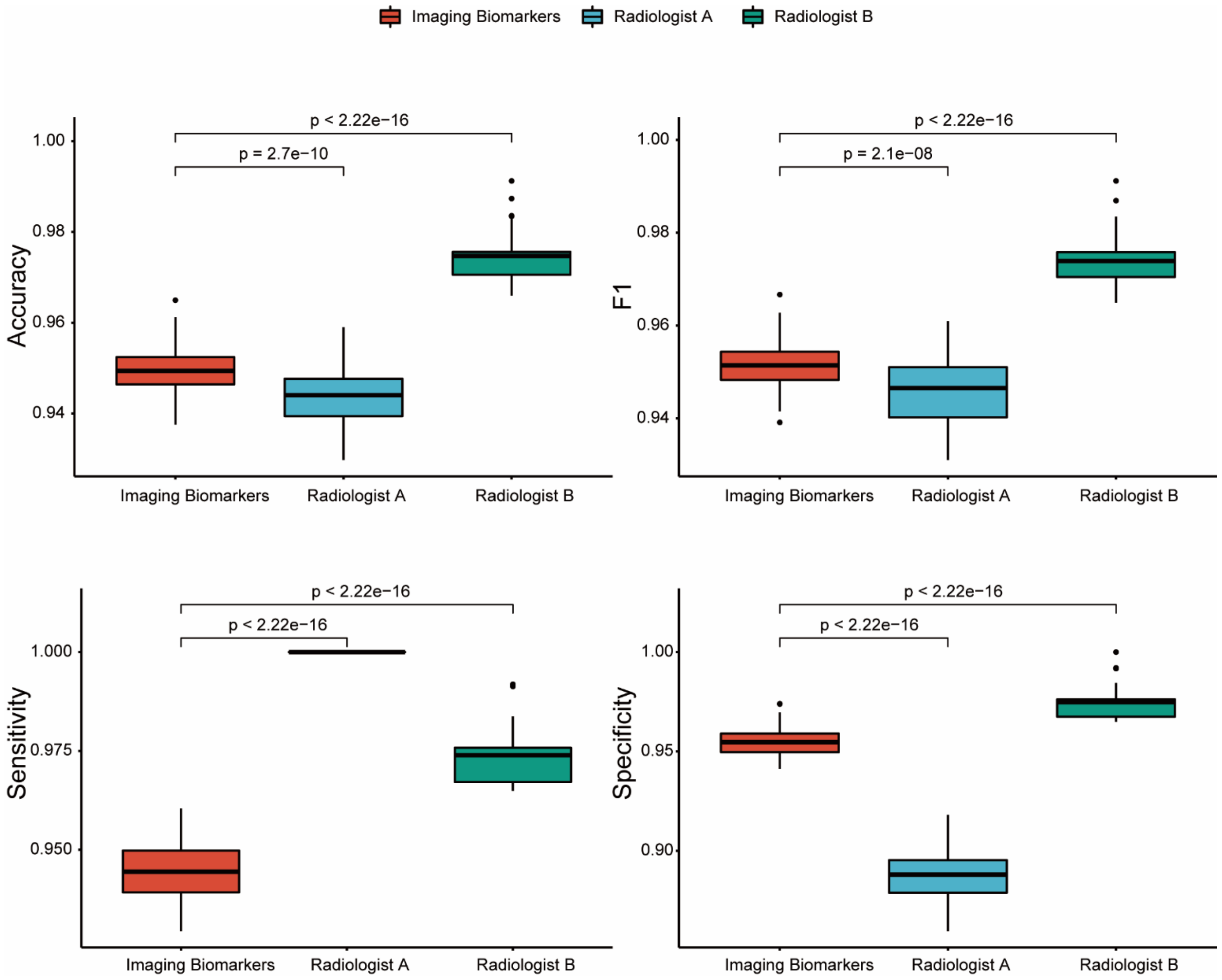

Supplementary Fig. 7 Statistical evaluation of screening performance between imaging biomarkers and two radiologists using bootstrapping strategy with 100 iterations and sampling rate of 80%.

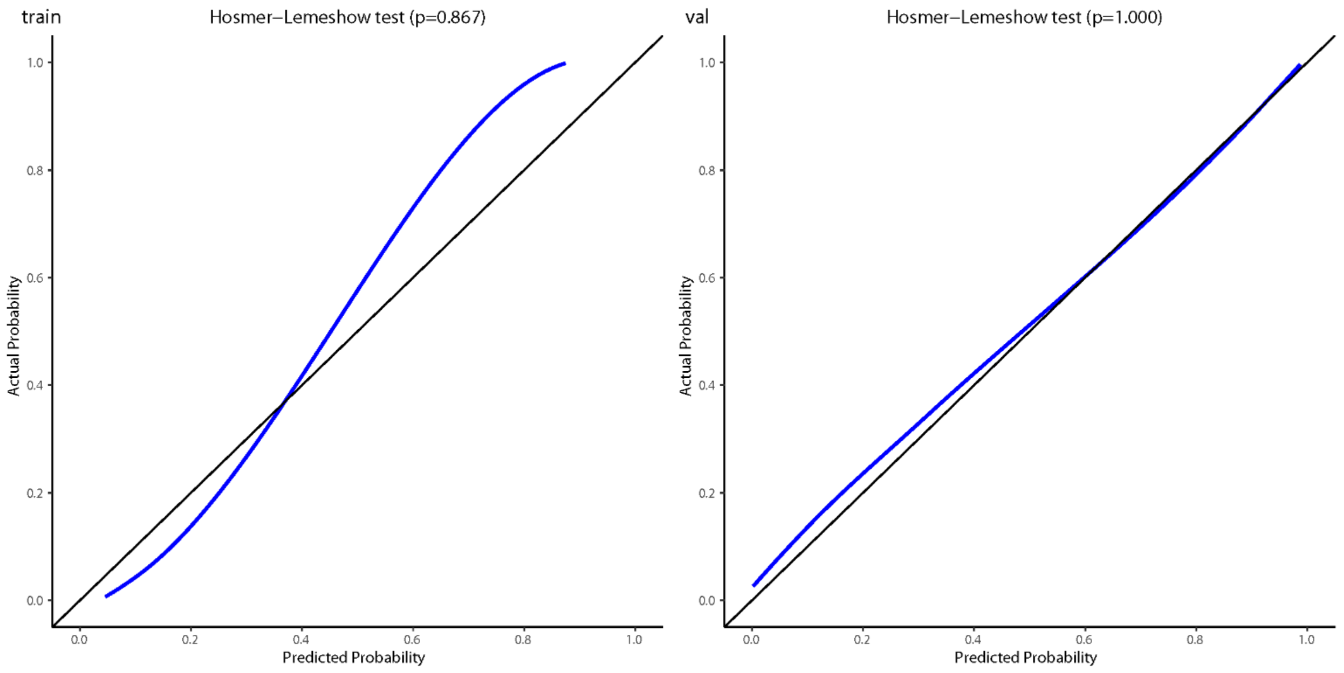

Supplementary Fig. 8 The Hosmer-Lemeshow test yielded a non-significant statistic on both training cohort (p = 0.867) and validation cohort (p = 1.000), which suggested that there were no departures of our screening model from perfect fit.

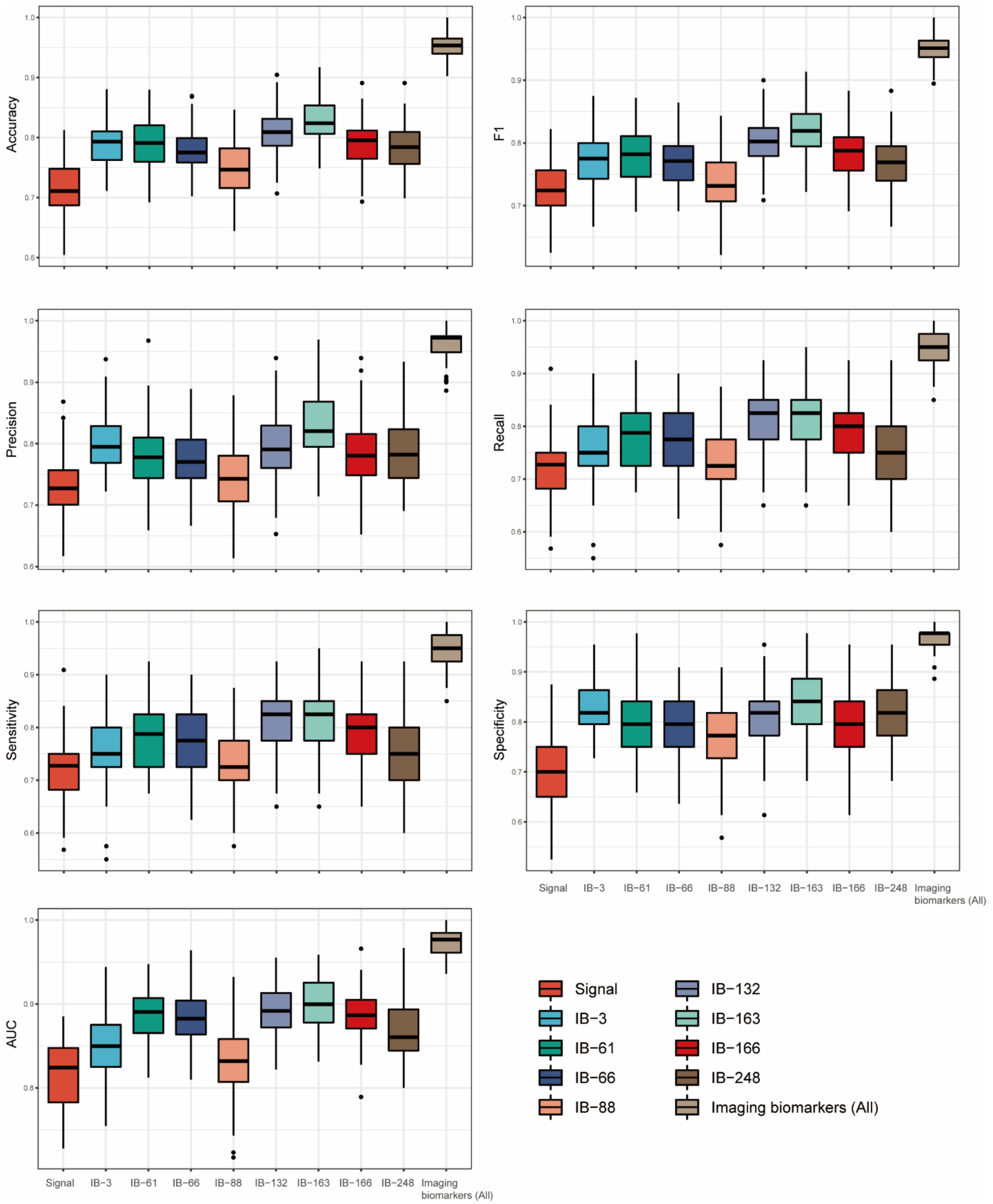

Supplementary Fig. 9 COVID-19 screening performance on the combination of both training and validation cohorts.

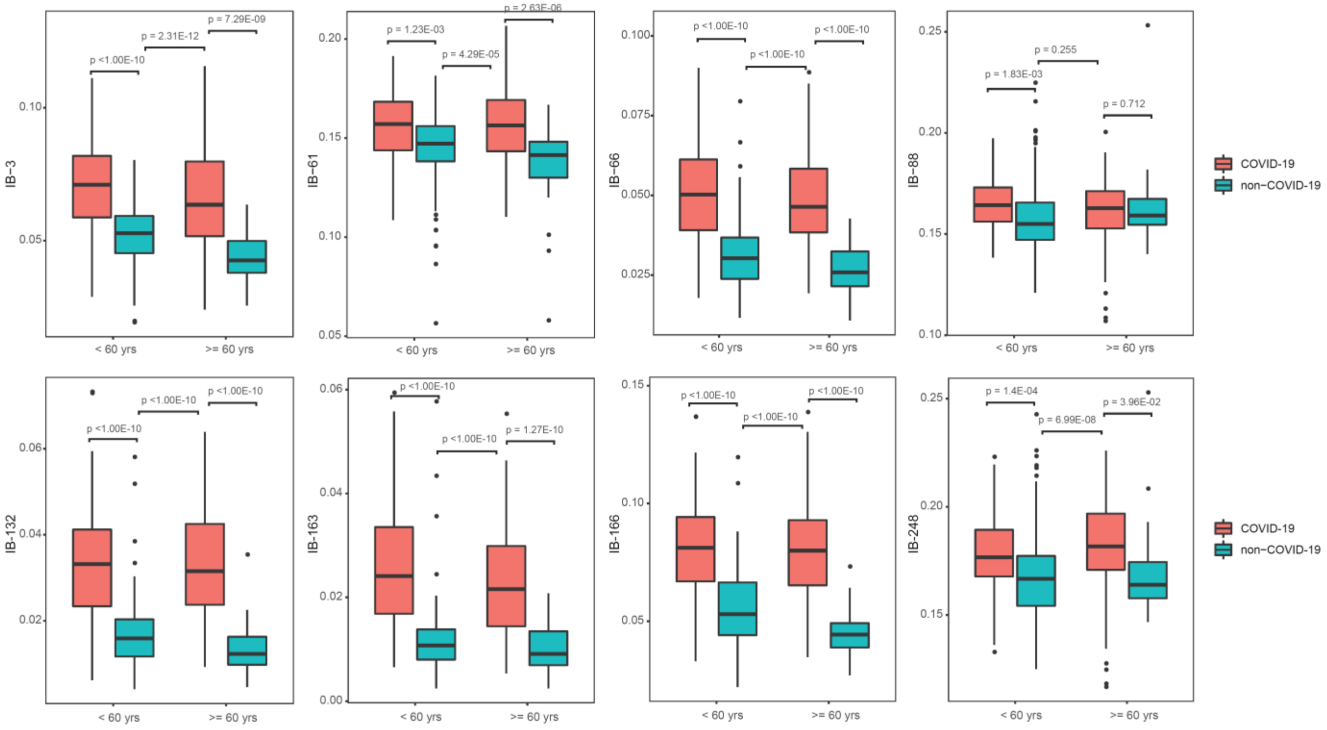

Supplementary Fig. 10 Abundance of each imaging biomarker across age groups in the combined cohorts demonstrates no age impact on imaging biomarkers.

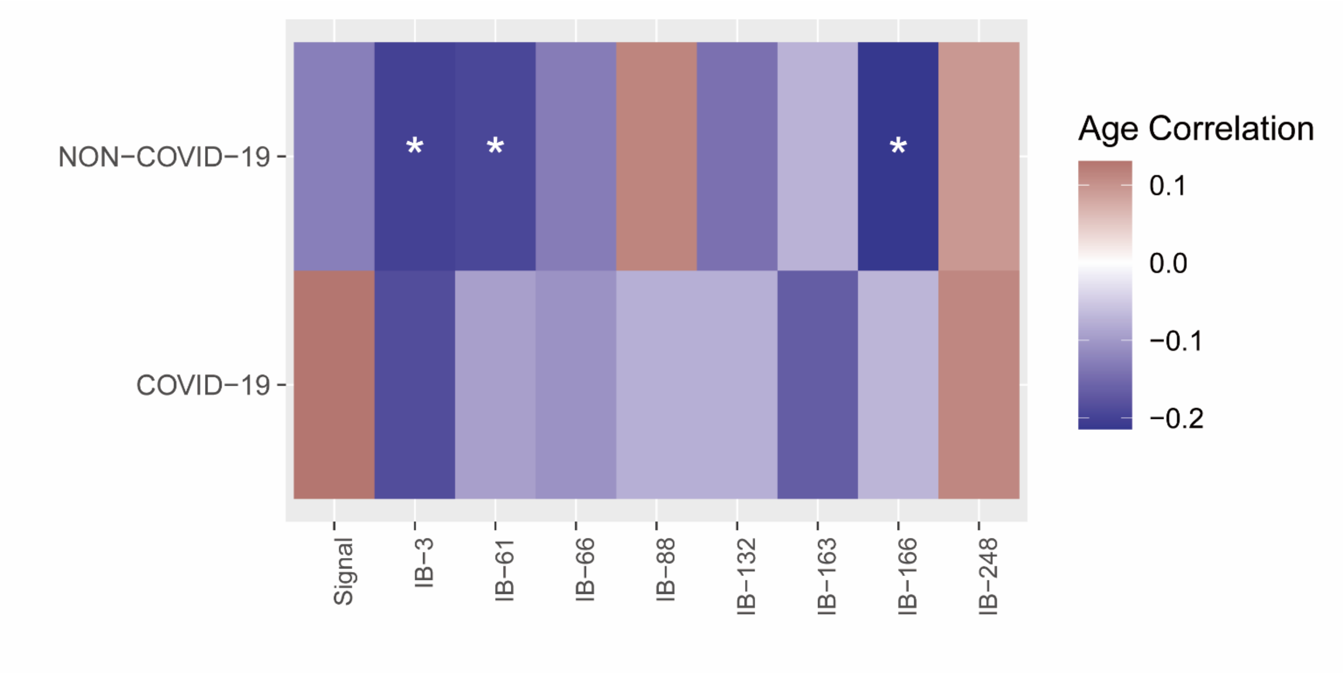

Supplementary Fig. 11 Spearman correlation of vasculature-like signal and individual imaging biomarker on combined cohorts (* indicates FDR < 0.05).

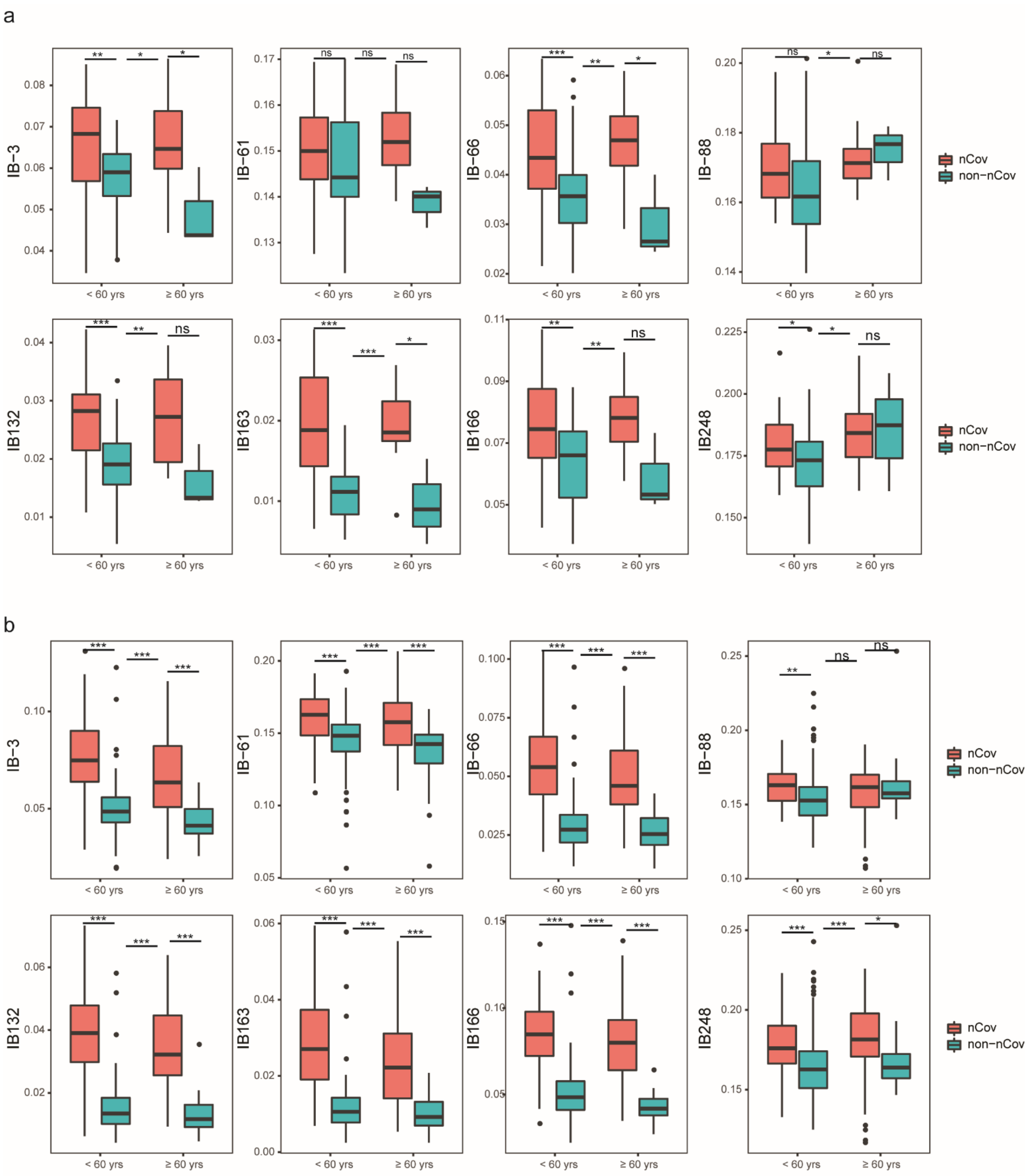

*** p < 0.001; ** p< 0.01; * p< 0.05

Supplementary Fig. 12 Abundance of each imaging biomarker across age groups in the training set (a) and validation set (b).

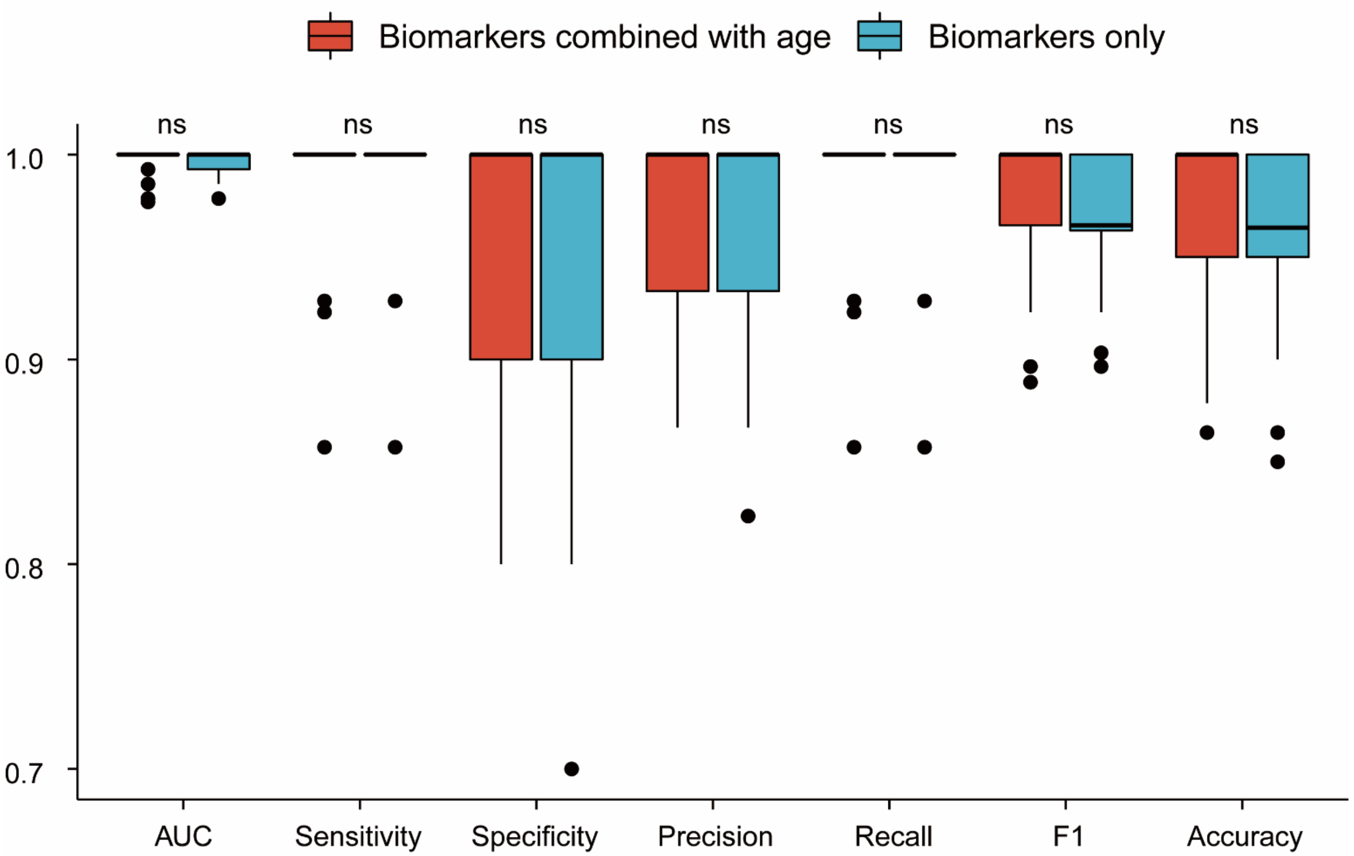

Supplementary Fig. 13 Performance comparison of prediction models on the training set with cross validation (100 bootstrap iterations, 80% training sample rate) based on significant imaging biomarkers and biomarkers combined with age (ns indicates non-significant: p-value >0.05).

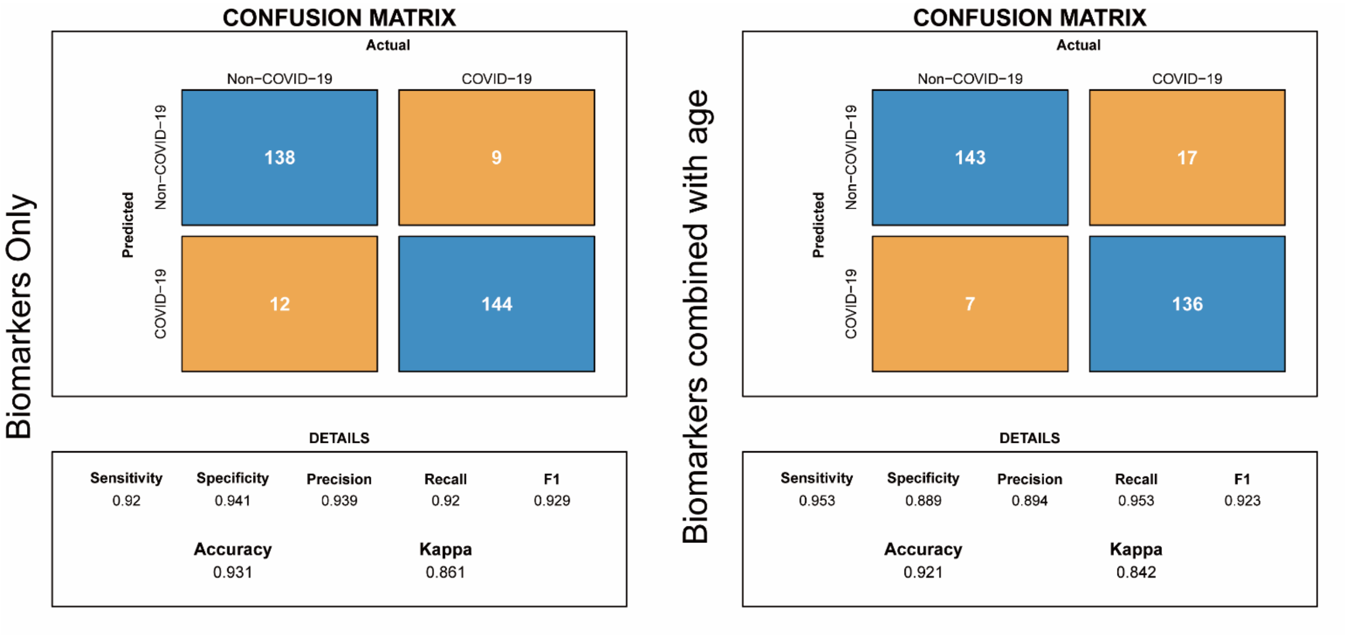

Supplementary Fig. 14 Performance comparison of prediction models on the validation cohort based on significant imaging biomarkers (left) and biomarkers combined with age (right).

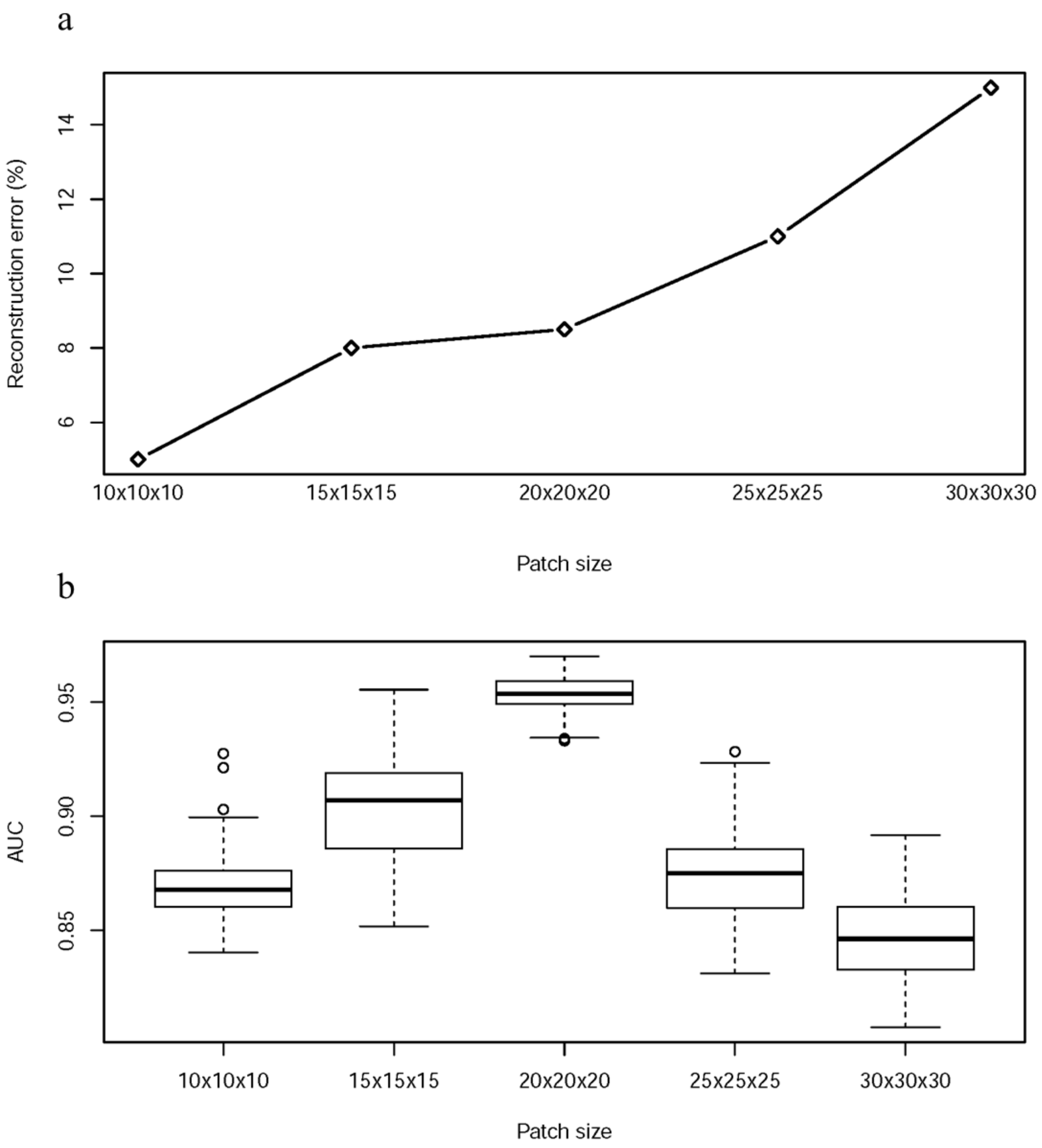

Supplementary Fig. 15 a. Reconstruction error across different patch sizes during unsupervised signature learning; **b.** cross-validation performance on training cohort (Hospital A) with signatures learned across different patch sizes**.**
