# Supplementary material for: Development and validation of chest CT-based imaging biomarkers for early stage COVID-19 screening
Source: Front Public Health. 2022 Sep 21;10:1004117. doi: 10.3389/fpubh.2022.1004117 (PMC9533142; doi:10.3389/fpubh.2022.1004117)
Supplement: Supplementary file 9 [file Table_3.docx]

Supplementary Table 3 COVID-19 screening performance on training cohort with vaculature-like signal, individual imaging biomarker and all imaging biomarkers

Feature	AUC	95%CI		Sensitivity	95%CI		Specificity	95%CI	
		Lower	Upper		Lower	Upper		Lower	Upper
Signal	0.721	0.536	0.861	0.600	0.400	0.900	0.857	0.571	1.000
IB-3	0.764	0.552	0.904	0.683	0.480	0.875	0.730	0.628	0.867
BI-61	0.873	0.753	0.979	0.750	0.572	1.000	0.824	0.686	1.000
IB-66	0.757	0.583	0.915	0.600	0.436	0.833	0.692	0.594	0.852
IB-88	0.871	0.719	0.961	0.750	0.522	0.953	0.800	0.632	0.926
IB-132	0.729	0.544	0.868	0.571	0.381	0.833	0.690	0.554	0.833
IB-163	0.893	0.764	0.986	0.750	0.583	1.000	0.818	0.692	0.926
IB-166	0.738	0.562	0.868	0.608	0.421	0.833	0.714	0.585	0.874
IB-248	0.870	0.710	0.992	0.750	0.563	1.000	0.800	0.675	0.966
Imaging Biomarkers (All)	1.000	0.982	1.000	1.000	0.860	1.000	1.000	0.875	1.000

Abbreviation: IB, Imaging biomarker; AUC, Area under curve; CI, Confidence interval
